# Machine Learning and Geospatial Modeling of Climate Change Impacts on Ethiopian Honeybees for Conservation and Resilient Agriculture

**DOI:** 10.1002/ece3.73842

**Published:** 2026-06-14

**Authors:** Diriba Tulu, Kalid Hassen Yasin, Tadele Bedo Gelete, Beyan Ahmed, Dinaol Belina

**Affiliations:** ^1^ School of Animal and Range Sciences, College of Agriculture and Environmental Sciences Haramaya University Dire Dawa Ethiopia; ^2^ Geo‐Information Science Program, School of Geography and Environmental Studies Haramaya University Dire Dawa Ethiopia; ^3^ School of Agricultural Economics and Agribusiness, College of Agriculture and Environmental Sciences Haramaya University Dire Dawa Ethiopia; ^4^ College of Veterinary Medicine Haramaya University Dire Dawa Ethiopia

**Keywords:** *Apis mellifera*, climate refugia, habitat fragmentation, machine learning

## Abstract

Global climate change is negatively impacting honeybee production and productivity, threatening survival, health, and pollination functions which are vital for agriculture and biodiversity. Thus, this study employed integrated machine learning and geospatial modeling (*Random Forest, Support Vector Machine, XGBoost*, and *LightGBM*) to predict current and future habitat suitability in Ethiopia under SSP2‐4.5 and SSP5‐8.5 (2041–2080), therefore promoting conservation and climate‐resilient agriculture. Variable importance analysis revealed that agro‐ecological zones were the most influential predictors, accounting for 14%–22% of the variance across models. Among bioclimatic factors, Bio19 (coldest quarter precipitation) emerged as a prominent driver (14.1% in RF; 10.3% in XGBoost), indicating the importance of dry‐season water availability. Model performance varied: Random Forest had the best predictive precision (specificity = 0.93); however, XGBoost better identified spatial clustering patterns. Under present conditions, Random Forest predicted 30.02% of the study area as highly suitable, especially in the Western Highlands, whereas LightGBM predicted 18.62%, showing increased habitat fragmentation. Forecasts for the future (considering only climate and static topography) indicate a significant reduction in highly suitable habitats, with a 46.2% decline under SSP5‐8.5 by the 2070s. Landscape‐level measurements indicated increased fragmentation, including a reduction in Shannon diversity (1.48–1.29) and a 19.2% increase in fractal dimension, indicating more complex patch topology. These findings recommended the need to restore pollinator corridors in highland refugia, promoting drought‐tolerant plants like *Vachellia abyssinica*, and integrating adaptive apiculture approaches.

## Graphical abstract

The current study used machine learning and geospatial analysis to predict how climate change will loosen and fragment suitable habitats for Ethiopian honeybees. Main factors like agro‐ecological zones and dry‐season precipitation were found to be critical for bee survival. The findings revealed an urgent need for targeted conservation strategies to protect pollination services and agricultural resilience.

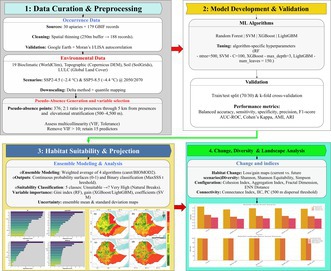

## 1 Introduction

Global warming is anticipated to increase average global temperatures by 1.5°C–4.8°C by 2100, posing a major threat to biodiversity and agroecosystems by changing species ranges and ecological interactions (IPCC 2018). Honeybees, which are important pollinators for both natural ecosystem functioning and agricultural productivity, are declining due to habitat degradation, pesticide introduction, and climate change‐induced phenological shifts (Rahimi et al. 2021; Sibaja Leyton et al. 2025). In tropical regions such as Ethiopia, these impacts are exacerbated by the narrow climatic tolerances of many endemic species, limited ecosystem buffering capacity, and a high dependence of rural livelihoods on pollinator‐mediated agricultural production shifts (Coallier et al. 2025; Gebremedhn et al. 2024).

Ethiopia, a biodiversity hotspot and Africa's biggest honey producer, is home to five subspecies of *Apis mellifera* , including the indigenous *Apis mellifera simensis* (Meixner et al. 2011). These honeybee populations maintain both ecological resilience and socioeconomic stability by providing important pollination services and sustaining the livelihoods of millions of people (FAO et al. 2021). Climate change scenarios for Ethiopia predict a temperature increase of 2°C–4°C, followed by increased rainfall variability, which is projected to impair pollinator habitats and reduce agriculture production and honey harvests (IPCC 2023). The present pace of climate change exceeds many species' adaptation capabilities, disrupting plant–pollinator relationships and destabilizing ecosystems. These effects are especially severe in tropical locations, where many species have already reached their top heat tolerance limits (Deutsch et al. 2008). In this context, there should be an urgent need for robust predictive modeling frameworks capable of characterizing habitat suitability for honeybee species under changing climatic conditions to design and implementation of evidence‐based conservation and management strategies.

Species Distribution Models (SDMs) have become an important tool in predicting the distribution and habitat suitability of different species under climate scenarios (Georgiades et al. 2023; Martín et al. 2021; Naimi and Araújo 2016). However, traditional correlative SDMs often undertake simplistic (often linear) species–environment relationships and overlook critical microhabitat heterogeneity and predictor interactions (Elith and Leathwick 2009). Machine learning algorithms overcome these limitations by integrally capturing complex non‐linear patterns and interactions (Lee‐Yaw et al. 2022; Phillips and Dudík 2008). Machine learning models have been demonstrated to be highly valuable in apicultural science, allowing crop yield prediction linked to pollinator services (Singh et al. 2025), bee behavior detection (Voudiotis et al. 2022), bee colony health detection, and honeybee production and harvest forecasting (Campbell et al. 2020; Ramirez‐Diaz et al. 2025). Spatial and agent‐based models further enhance these applications by simulating hive dynamics and forage resource availability (Sahin Demirel 2024). Despite these advances, a major gap remains because most ML‐based pollinator SDMs rely on coarse‐resolution bioclimatic data, which are less able to capture landscape‐scale features necessary for microhabitat suitability (Clarke and Robert 2018; Coallier et al. 2025; Ma'moun et al. 2025; Rahimi et al. 2021). Applications to endemic African honeybee species under climate change remain scarce, with existing research often based on historical distributions and broad ecological suitability rather than locally specific environmental factors (Assefa and Lemma 2022). While ML has been used to model pollinator declines elsewhere (e.g., bumblebees: Krechemer and Marchioro 2020; Sirois‐Delisle and Kerr 2018; honeybee range shifts: Lima and Marchioro 2021; MacInnis et al. 2023; Ma'moun et al. 2025; Rahimi et al. 2021; Tennakoon, Apan, and Maraseni 2024), limited study has applied ensemble ML approaches to project climate change impacts on *A. mellifera* in Ethiopia. Previous Ethiopian honeybee suitability studies utilized GIS with the Analytic Hierarchy Process and maxent model (Assefa and Lemma 2022) or basic SDMs, lacking the predictive power, uncertainty quantification, and ability to handle complex variable interactions inherent in advanced ensemble ML approaches. Moreover, no research quantifies future habitat fragmentation or identifies climate refugia for this socioeconomically crucial species using spatially explicit, high‐resolution data with different ML algorithms under different climate change scenarios.

This study develops an ensemble machine learning framework combining four state‐of‐the‐art algorithms such as Random Forest (RF), Support Vector Machines (SVM), Extreme Gradient Boosting (XGBoost), and Light Gradient Boosting Machine (LightGBM) with geospatial data to model current and future habitat suitability for *A. mellifera* across Ethiopia under SSP2‐4.5 and SSP5‐8.5 (2041–2080). The approach integrated non‐climatic predictors (soil, topography) with downscaled bioclimatic variables, computes habitat fragmentation using landscape metrics, and identifies microclimatic refugia. Because, habitat loss and fragmentation across scales directly affect species abundance, diversity, and productivity, these analyses can guide optimal apiary placement (Hackett et al. 2024). The study aimed to (i) model current and future habitat suitability using ensemble ML, (ii) identify key climatic and non‐climatic drivers of *A. mellifera* distribution, and (iii) assess climate‐driven habitat fragmentation and connectivity loss. The findings will support targeted conservation actions such as establishing pollinator corridors and developing drought‐resilient forage species aligned with Ethiopia's Climate‐Resiient Green Economy Strategy and SDGs on food security, climate action, and biodiversity conservation (UN 2024).

## 2 Materials and Methods

### 2.1 Description of the Study Area

**FIGURE 1:**
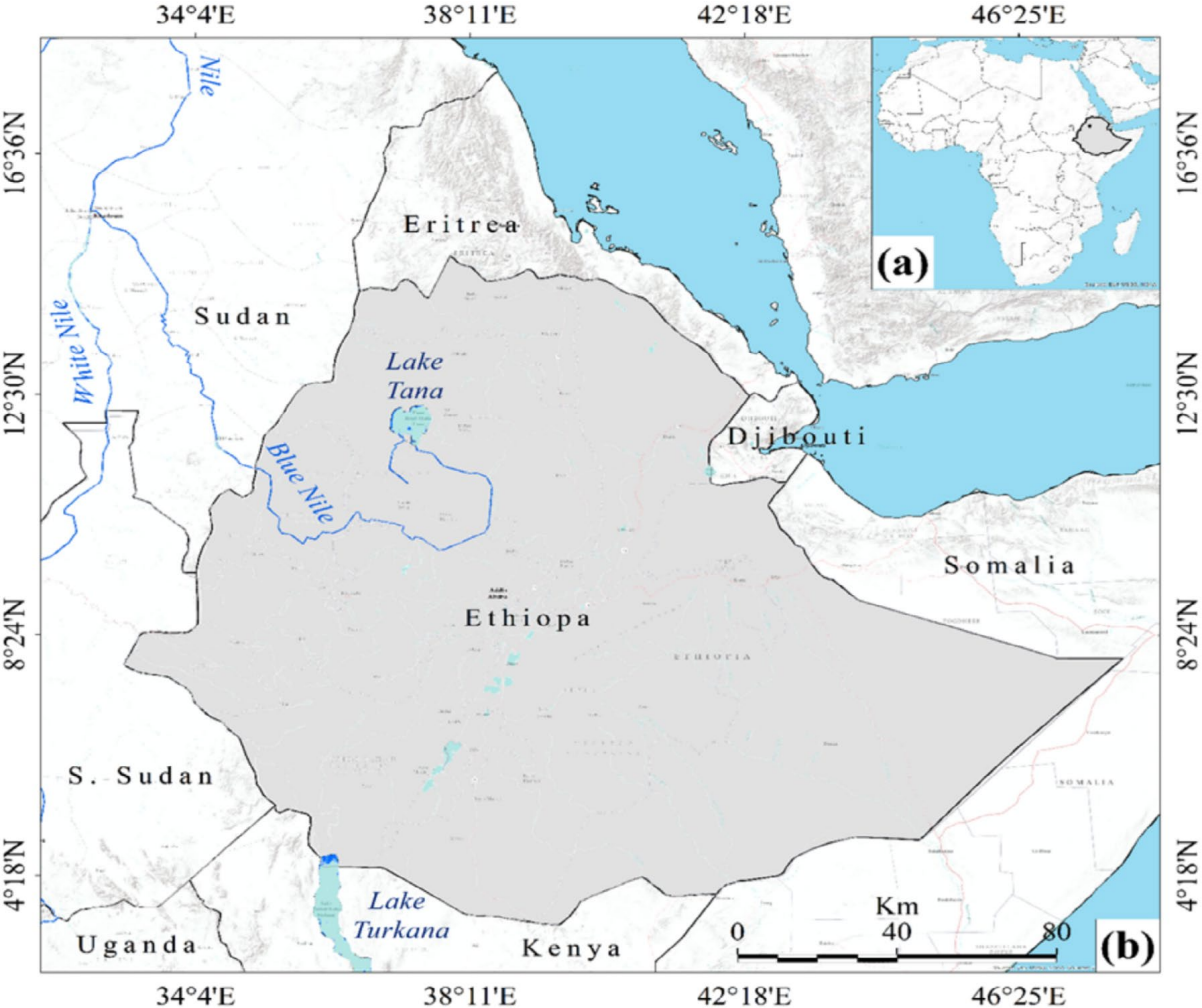
Map of the study area. (a) Africa, (b) Ethiopia.

Ethiopia is located in the tropics (3°–15° N, 33°–48° E) and covers approximately 1.1 million km^2^. The topography varies from 116 a.s.l. in the Danakil Depression to 4620 a.s.l. in Ras Dejen (see Figure 1). This variety has given rise to 11 Afrotropical ecoregions, which have been designated as a Global 200 priority for biodiversity conservation (NaBU 2020; Olson and Dinerstein 2002). The nation is divided into nine agroecological zones (AEZs), with tropical‐cool/subhumid accounting for 44.59%, tropical‐warm/arid for 18.77%, and tropical‐cool/semiarid for 15.06% (Gashaw et al. 2023). Climate varies significantly, with highland AEZs experiencing low minimum temperatures (5.03°C–6.67°C) and substantial rainfall (up to 1368 mm), whereas lowlands reach 31°C with as little as 379 mm per year (Asefa et al. 2020). These gradients affect vegetation, water availability, and agriculture. Importantly, highland AEZs, particularly tropical‐cool/humid, subhumid, and semiarid zones, are the primary habitats of the endemic honeybee *A. m. simensis* , which prefers cooler, florally rich environments (Atsbha et al. 2025).

### 2.2 Methodological Framework

**FIGURE 2:**
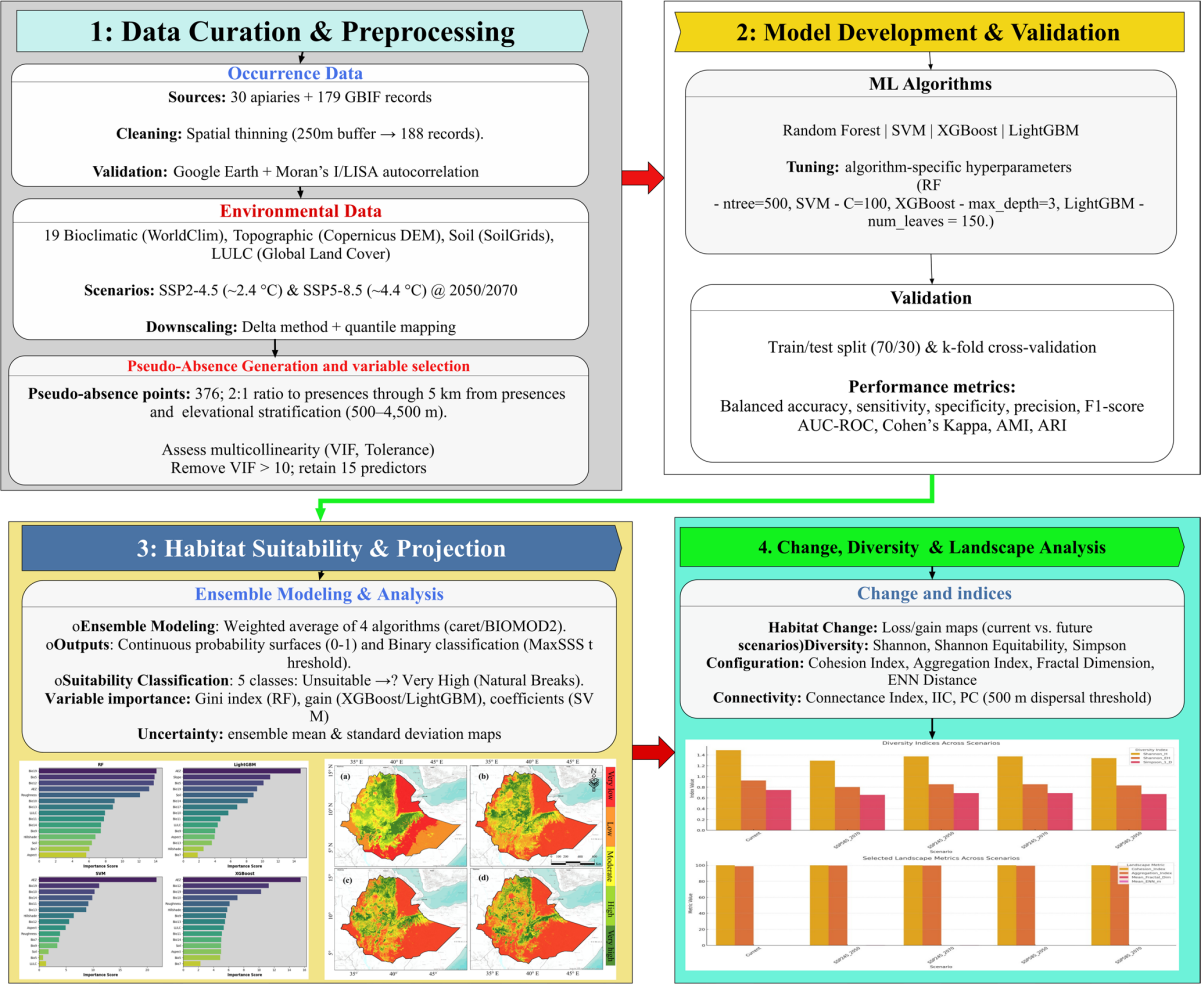
Methodological framework of the study.

This study implemented the Open Data Protocol for Automated Modeling of Species Distributions (ODMAP) (Zurell et al. 2020) to safeguard methodological transparency and reproducibility throughout the species distribution modeling process. The systematic work flow developed chronologically through five important stages (Figure 2): (1) data acquisition and preprocessing of *A. mellifera* occurrence records through spatial validation and thinning procedures; (2) careful selection and processing of environmental predictors, including bioclimatic, topographic, edaphic, and land‐use variables; (3) calibration and validation of four machine learning algorithms (Random Forest, Support Vector Machine, XGBoost, LightGBM) using spatially partitioned data; (4) projection of habitat suitability under current and future climate scenarios; and (5) comprehensive spatial analysis of suitability patterns and landscape fragmentation metrics. The modeling approach exclusively utilized combined climatic‐environmental predictors to capture synergistic interactions between broad‐scale climate patterns and localized landscape factors. Future projections were generated using two Shared Socioeconomic Pathways—SSP2‐4.5 (moderate emissions pathway, ~2.4°C warming by 2100) and SSP5‐8.5 (high emissions pathway, ~4.4°C warming by 2100)—evaluated at mid‐century (2041–2060) and late‐century (2061–2080) time horizons (IPCC 2021).

### 2.3 Honeybee Occurrence Data Collection and Processing

Presence records for *A. mellifera* in Ethiopia were compiled from two sources: (1) georeferenced managed apiaries (*n* = 30) documented in agro‐ecological studies (Bayissa et al. 2024; Demis et al. 2025; Gebrehiwet et al. 2019; Hunde et al. 2023; Tilahun et al. 2022), and (2) coordinate points of occurrences (*n* = 179) downloaded from the Global Biodiversity Information Facility (GBIF) on February 13, 2025 GBIF records were temporally filtered (1990–2025) to align with available climate data (GBIF 2025). Museum specimens were excluded due to potential biases in historical distribution representation (Araújo and New 2007). All records were geospatially verified with Google Earth Pro v.7.3, which evaluated coordinate accuracy against high‐resolution images and manually fixed unclear spots. To decrease spatial autocorrelation and satisfy Ethiopia's Biosecurity Act apiary spacing criteria (MoA 2024)., a minimum intercedes distance of 250 m (matching the resolution of environmental covariates) was enforced using ArcGIS 10.8 and the R package spin (Aiello‐Lammens et al. 2015). The dataset was reduced from 209 to 188 independent recordings (10.05% decrease), showing well‐managed apiaries and increasing robustness for ecological niche modeling. Residual spatial autocorrelation was measured using Global Moran's I (Bivand et al. 2013), which demonstrated little geographical dependency, while Local Indicators of Spatial Association (LISA) (Anselin 1995) revealed no notable clustering or outliers.

### 2.4 Environmental Predictors

#### 2.4.1 Selection and Preprocessing of Environmental Variables

The predictor variables were selected based on their mechanistic associations with *A. m. simensis* ecology, including thermoregulatory limitations, floral resource dependence, nesting site specificity, and anthropogenic stress tolerance (Bayissa et al. 2024; Coallier et al. 2025; Lima and Marchioro 2021; Ma'moun et al. 2025; Tabor and Koch 2021; Tennakoon, Apan, and Maraseni 2024; Zapata‐Hernández et al. 2024) (Table S3). Nineteen bioclimatic variables for the historical baseline period (1970–2000) were obtained from the WorldClim v2.1 database (https://www.worldclim.org/) at a spatial resolution of 30 arc‐seconds (~1 km^2^) (Fick and Hijmans 2017). Topographic variables, including elevation, slope, aspect, and terrain roughness, were derived from the Copernicus Digital Elevation Model at 30 m resolution (https://scihub.copernicus.eu/) (AIRBUS 2022). Terrain analyses were conducted using the terrain () function within the raster package in R version 4.2.2 (R Core Team 2025).

Soil variables, such as pH, organic carbon, and texture fractions (sand, silt, clay), were extracted from ISRIC SoilGrids v2.0 at 250 m resolution, resampled to 1 km, and retained based on their influence on floral community structure and nectar/pollen productivity. Land use and land cover (LULC) data were sourced from the Global Land Cover Product (30 m resolution). Due to the lack of Ethiopia‐specific high‐resolution maps capturing smallholder agroforestry, grazing mosaics, and traditional farming systems, the global LULC data were validated against Sentinel‐2 imagery, ground‐truth GPS points, and auxiliary datasets from FAO Africover (FAO and IIASA 2022). Derived indices of seasonal cropland flowering dynamics were incorporated based on known crop calendars, MODIS Enhanced Vegetation Index (EVI), and regional agricultural cycles. Data on Ethiopian agro‐ecological zones were obtained from the Ethiopian National Agri‐Data Hub and cross‐validated using datasets from the Food and Agriculture Organization (2020). All spatial layers were projected to WGS 1984 (EPSG:4326), clipped to Ethiopia's extent (3°40′–15° N, 33°–48° E), and uniformly resampled to a 1 km^2^ resolution using bilinear interpolation, following standard geospatial data processing protocols (Hijmans and van Etten 2012).

### 2.5 Climate Change Projections and Downscaling Procedures

Climate projections were obtained from the Coupled Model Intercomparison Project Phase 6 (CMIP6) using the HadGEM3‐GC31‐LL General Circulation Model (GCM), selected for its high accuracy in simulating regional climate variability in Eastern Africa, particularly in Ethiopia (Brands et al. 2013). Two Shared Socioeconomic Pathways (SSPs) were used: SSP2‐4.5 (moderate mitigation and adaptation) and SSP5‐8.5 (high emissions with minimal policy intervention), indicating anticipated global warming of ~2.4°C and ~4.4°C by 2100, respectively (Knutti et al. 2017). The HadGEM3‐GC31‐LL model was chosen for its strong performance in Africa and Europe (Brands et al. 2013), and both SSPs were chosen to capture moderate and extreme warming trajectories relevant for assessing endemic species vulnerability (Legesse 2016). Downscaled climate data for mid‐ to late‐century periods were collected from WorldClim v2.1 (https://www.worldclim.org/future). Downscaled climate data for mid‐ to late‐century periods were collected from WorldClim v2.1 (Fick and Hijmans 2017).

### 2.6 Predictor Selection for Current Versus Future Projections

After multicollinearity filtering (see Section 2.4), all 15 environmental predictors (Bio1, Bio5, Bio7, Bio9, 14, Bio19), topographic variables (elevation, slope, aspect, hillshade), soil properties (organic carbon, pH, texture), land use/land cover, and agro ecological zones were retained for modeling current habitat suitability. For future forecasts (2041–2060 and 2061–2080 under SSP2.4.5 and SSP5.8.5), a reduced number of bioclimatic variables and static topographic predictors (elevation, slope, aspect, hillshade) was utilized. Soil properties, land use/land cover, and agro ecological zones were excluded for three reasons: (i) no validated future projections exist for Ethiopia at 1 km resolution; (ii) AEZs are defined by current climate, making their use in climate projection circular (Sutherst et al. 2007); and (iii) holding dynamic LULC constant would introduce unrealistic assumptions given Ethiopia's rapid land use change (Aerts et al. 2019). Topographic variables were preserved since they change over geologic (rather than decadal) timescales, as is customary in climate‐driven SDMs (Austin and Van Niel 2011; Franklin 2010).

### 2.7 Generation of Pseudo‐Absence Data

In the absence of validated *A. mellifera* records, pseudo‐absence data were produced using ecologically informed approaches to eliminate geographic and environmental bias (Wang et al. 2023). Using the dismo R package's randomPoints() function, 376 pseudo‐absence points (2:1 ratio to presence data) were generated. Points were tested outside a 5 km buffer surrounding known occurrences and limited to appropriate ecoregions, excluding metropolitan areas, bodies of water, and extreme climate zones (Wisz and Guisan 2009). Mahalanobis distance filtering eliminated places beyond the species' environmental envelope, whereas stratification across 500–4500 m elevation assured coverage of the species' distribution range and decreased sample bias (Etherington 2021).

### 2.8 Multicollinearity Assessment and Variable Selection

To validate model robustness, multicollinearity among predictors was measured using the variance inflation factor (VIF) and tolerance measures. The VIF calculates the degree to which a predictor's variance is inflated due to linear relationships with other variables, as follows:

$${\mathrm{VIF}}_{i}=\frac{1}{1-{R_{i}}^{2}}$$

where $R_{i}^{2}$ is the coefficient of determination from regressing the *i*th predictor against all others, and tolerance, defined as the reciprocal of VIF (Tolerance = 1/VIF), measures the proportion of variance in a predictor not explained by others (Dormann et al. 2013).

Variables with VIF > 10 (Tolerance < 0.2) were removed successively to decrease redundancy, following ecological modeling best practices (Dormann et al. 2013). This restrictive criterion resulted in maintained predictors with minimal collinearity while keeping ecologically important features. The final collection included 15 predictors: bioclimatic indicators (e.g., yearly mean temperature [Bio1], precipitation in the driest quarter [Bio17]), topographic variables (elevation, slope), and soil characteristics (organic carbon content). These parameters were evaluated according to their known influence on *A. mellifera* foraging behavior, colony survival, and nesting habitat suitability (Hijmans and van Etten 2012). These predictors showed the important factors of habitat suitability for *A. mellifera* under both present and future environmental conditions.

### 2.9 Modeling Algorithms and Evaluation

An ensemble machine learning framework was used to analyze the ecological niche and predict habitat suitability for *A. mellifera* in Ethiopia under present and future climatic scenarios. Given ecological complexity, nonlinear biotic‐abiotic interactions, and scarce, spatially skewed occurrence data, model selection emphasized both predictive performance and ecological interpretability (Dormann et al. 2013; Elith and Leathwick 2009). Four algorithms were used: Random Forest (RF), Support Vector Machines (SVM), XGBoost, and LightGBM. These were chosen because of their high performance in species distribution modeling, ability to handle high‐dimensional environmental data, reduce overfitting, and capture complex non‐linear relationships (El Alaoui and Idri 2024; Hao et al. 2019). Using many techniques enhances robustness and provides ecologically significant estimates of species–environment correlations, especially for *A. mellifera* , which is sensitive to microclimate and seasonal variations.

#### 2.9.1 Random Forest (RF)

RF is a bagging‐based ensemble technique that generates many decision trees from bootstrap samples and combines their predictions using majority elective or averaging (Breiman 2001). It is ideal for ecological modeling because it can handle collinear predictors, non‐linear interactions, and imbalanced datasets (Cutler et al. 2007). In the case of *A. mellifera* , RF was particularly useful in identifying the most relevant bioclimatic factors, such as temperature seasonality (Bio4) and driest quarter precipitation (Bio17). The significance of predictors was evaluated using permutation‐based metrics such as the Mean Decrease in Accuracy, which improves ecological interpretability (Gregorutti et al. 2017). Model tuning included 500 trees (ntree = 500) and the number of variables sampled at each node (mtry) set to the square root of the total number of predictors (Probst et al. 2019). RF also demonstrated resilience to spatial biases inherent in species occurrence records, making it suitable for datasets with clustered sampling typical of biodiversity data in Ethiopia.

#### 2.9.2 Support Vector Machine (SVM)

SVM is a kernel‐based learning algorithm that constructs hyperplanes to separate data classes in high‐dimensional feature spaces (Vapnik 1999). By employing a radial basis function (RBF) kernel, SVM effectively models non‐linear relationships between species occurrence and environmental variables. This is critical for *A. mellifera* , whose ecological tolerances exhibit sharp thresholds. For example, colony collapse risk increases dramatically under extreme heat or low humidity conditions (Leroy et al. 2016). SVM's capacity to model such threshold effects enhances its utility in ecophysiological modeling. The regularization parameter (*C*) was set to 100 to balance margin maximization with misclassification tolerance, and gamma (*γ*) was fixed at 0.1 to control the kernel's complexity. These parameters allowed for capturing the complex bioclimatic gradients characteristic of Ethiopia's highland‐lowland transitions and agroecological zones (Drake et al. 2006).

#### 2.9.3 Extreme Gradient Boosting (XGBoost)

XGBoost is a tree‐based boosting algorithm that iteratively minimizes prediction errors through gradient descent optimization (Chen and Guestrin 2016). It builds additive models by sequentially fitting new trees to the residuals of prior predictions, enabling the capture of subtle and cumulative effects of climatic trends, such as slight increases in temperature (Bio1) or shifts in precipitation seasonality (Bio15). XGBoost incorporates regularization through both L1 (alpha = 0.1) and L2 (lambda = 1) penalties, which helps mitigate overfitting, especially important in datasets with limited or biased occurrence points. For this study, hyperparameters were tuned to max_depth = 3 and learning_rate = 0.1, achieving an optimal balance between model complexity and generalization (Zheng et al. 2017). XGBoost was particularly effective in modeling narrow ecological niches and rare presence events of *A. m. simensis* in regions with abrupt topoclimatic gradients.

#### 2.9.4 Light Gradient Boosting Machine (LightGBM)

LightGBM is a gradient boosting algorithm that uses histogram‐based learning and optimizations such as Gradient‐based One‐Side Sampling and Exclusive Feature Bundling to reduce computational overhead (Ke et al. 2017). LightGBM was especially advantageous for modeling at high spatial resolutions due to its speed and memory efficiency, enabling the incorporation of fine‐grained topographic and edaphic predictors. The algorithm uses a leaf‐wise tree growth strategy, prioritizing splits with the highest information gain. This makes it well‐suited for identifying marginal habitats, where small changes in environmental conditions such as ±1°C variation in Bio10 (mean temperature of warmest quarter) can significantly alter foraging activity or brood development (Potts et al. 2016). LightGBM was tuned with num_leaves = 150 and feature_fraction = 0.8 to reduce overfitting while retaining model responsiveness to subtle bioclimatic signals (Jiang et al. 2022).

### 2.10 Model Validation and Performance Assessment

To provide reliable ecological inference, four machine learning algorithms (RF, SVM, XGBoost, and LightGBM) were tested utilizing a multi‐method validation framework. The dataset was divided into 70% training and 30% validation sets using random sampling, which is a popular method in ecological modeling due to its simplicity and transparency (Allouche et al. 2006). In addition, k‐fold cross‐validation was used to prevent overfitting and increase model dependability (Manes et al. 2006). Algorithm performance was quantified using threshold‐dependent metrics (balanced accuracy, sensitivity [recall], specificity, precision, F1‐score) derived from confusion matrices (true positives [TP], true negatives [TN], false positives [FP], false negatives [FN]) and threshold‐independent metrics such as the Area Under the Receiver Operating Characteristic Curve (AUC‐ROC), which evaluates the trade‐off between true positive rate and false positive rate across classifications. To assess spatial consistency and classification reliability, agreement metrics (Cohen's Kappa, allocation disagreement, quantity disagreement) and cluster similarity indices (Adjusted Mutual Information [AMI], Adjusted Rand Index [ARI]) were calculated to ensure alignment between predicted and observed *A. mellifera* distributions (Table S2). To ensure repeatability, model influences were initialized with a fixed random seed, and hyperparameters (e.g., tree depth, learning rate) were optimized using grid search, using standardized techniques for species distribution modeling (Zurell et al. 2020).

### 2.11 Feature Importance and Uncertainty Quantification

The value of predictor variables was assessed using algorithm‐specific measures, including mean Gini index reduction (RF; Breiman 2001), gain importance (XGBoost/LightGBM; Chen and Guestrin 2016; Ke et al. 2017), and coefficient weights (SVM). Uncertainty was measured by averaging ensemble mean and standard deviation maps across algorithms to highlight areas of strong consensus or variability (Araújo and New 2007). This approach improves prediction robustness and provides spatially explicit guidance for conservation prioritization by highlighting areas that require adaptive management or additional data (Guisan et al. 2013).

#### 2.11.1 Ensemble Model

Ensemble modeling combines predictions from multiple models to improve prediction reliability and accuracy, leveraging their strengths to outperform single‐model approaches (Evgeniou and Pontil 2001). RF provides interpretability through feature importance scores, while LightGBM offers computational efficiency (Breiman 2001; Chen and Guestrin 2016). SVM effectively detects ecological thresholds, and XGBoost refines accuracy via iterative gradient boosting (Chen and Guestrin 2016). This integration allows the ensemble to model both broad habitat suitability patterns and fine‐scale environmental responses for *A. mellifera* , critical for conservation and climate‐adaptive apiculture. Using the caret Ensemble package in R (Deane‐mayer 2024). Predictions from four models were aggregated using a weighted average technique that is based on individual model evaluation metrics. BIOMOD2 and caret packages in R facilitated species distribution modeling (Thuiller et al. 2009). The same ensemble weighting approach was used for future forecasts, but only model predictions based on the reduced predictor set (climate and topography).

### 2.12 Geospatial Habitat Suitability and Change Analysis

Habitat suitability for *A. mellifera* was represented by continuous probability (0–1), with values around 1 indicating ideal environmental conditions (Goulson et al. 2015). The Natural Breaks (Jenks) technique in ArcGIS Pro was used to categorize the probabilities as inappropriate (< 0.10), low suitability (0.10–0.28), moderate appropriateness (0.28–0.48), high suitability (0.48–0.69), and very high suitability (> 0.69) classes according to established guidelines (Jha et al. 2022; Yousefi et al. 2023; Zhang et al. 2019). ArcGIS 10.8 was used to generate suitability layers, with areas quantified through pixel‐based analysis. Thresholds were chosen to maximize sensitivity and specificity, resulting in biologically significant delineations of suitability under present and future climatic scenarios (Liu et al. 2013). To assess habitat changes for *the species*, these continuous maps were further converted into binary presence–absence outputs using the MaxSSS (maximum training sensitivity plus specificity) threshold, which effectively balances omission and commission errors to enhance model accuracy (Saupe et al. 2019; Taubert et al. 2018). Habitat loss was characterized as regions that are now appropriate becoming unsuitable under future warming scenarios (SSP2‐4.5 and SSP5‐8.5 for 2050 and 2070), whereas habitat gain refers to new areas becoming suitable. These dynamics were evaluated with changes in mean suitability values, showing that an overall decrease in habitat suitability is a critical signal of extinction risk, particularly for range‐restricted and fragile bird species (Aligaz et al. 2024; BirdLife International 2022; Bladon et al. 2021).

### 2.13 Habitat Diversity and Landscape Configuration Analysis

Landscape metrics were evaluated from binary habitat rasters using R v4.2 (R Core Team 2025). The vegan package was used to quantify habitat diversity using the Shannon Diversity Index (*H*′), Shannon Equitability (*E*_h_), and Simpson Index (1‐D), which measure richness, distribution evenness, and dominance patterns (Oksanen et al. 2022). Landscape metrics (Hesselbarth et al. 2019), employing FRAGSTATS‐compatible algorithms to compute the Cohesion Index (physical habitat connectivity), Aggregation Index (patch clustering intensity), Mean Fractal Dimension (shape complexity; 1–2 scale), and Mean Euclidean Nearest Neighbor Distance (patch isolation in meters) were used to analyze spatial configuration, with FRAGSTATS‐compatible algorithms calculating the Cohesion Index (physical habitat connectivity), Aggregation Index (patch clustering intensity), Mean Fractal Dimension (shape complexity; 1–2 scale), and Mean Euclidean Nearest Neighbor Distance (patch isolation in meters). Functional connectivity was evaluated using graph‐theoretic approaches in igraph and gdistance (van Etten 2017), calculating the Connectance Index (functional links proportion), Integral Index of Connectivity (IIC) (area‐weighted connectivity), and Probability of Connectivity (PC) (interaction likelihood) with a biologically parameterized 500‐m dispersal threshold based on empirical *A. m. simensis* foraging ecology (Ejigu et al. 2021). Spatial visualizations were generated using ggplot2 (Wickham 2016).

## 3 Results

### 3.1 Multicollinearity and Variable Importance in Habitat Suitability Modeling

**FIGURE 3:**
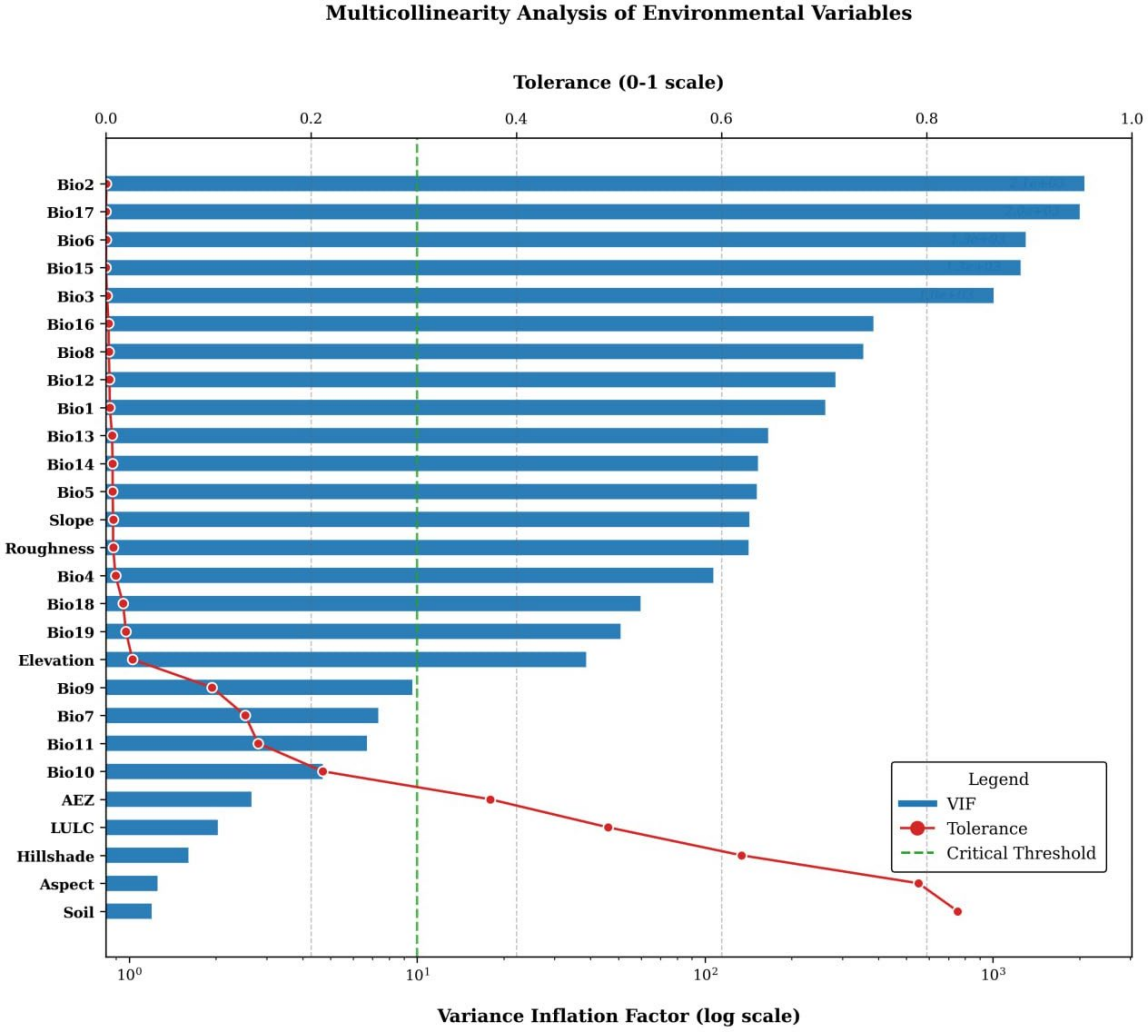
Multicollinearity (VIF and tolerance) in habitat suitability modeling of *Apis mellifera*.

Based on multicollinearity analysis, temperature‐related predictors, such as Bio3 (VIF = 1012.1), Bio6 (VIF = 1325), and Bio2 (VIF = 910.2) showed extreme collinearity (tolerance < 0.001), and as well as the precipitation seasonality variables Bio15 and Bio17 (VIF = 564) (Figure 3). Additionally, topographic features, such as slope and Roughness (VIFs > 140), displayed similar redundancy. In contrast, 15 variables exhibited acceptable multicollinearity (VIF ≤ 10; tolerance > 0.10). Retained bioclimatic predictors include Bio1, Bio5, Bio7, Bio9, Bio10, Bio11, Bio12, Bio13, Bio14, and Bio19, covering key temperature and precipitation gradients. Non‐climatic variables such as AEZ, Soil, Aspect, LULC, and Hill shade showed minimal collinearity (VIF < 3), supporting their stability model, reducing redundancy, and maintaining ecological interpretability.

**FIGURE 4:**
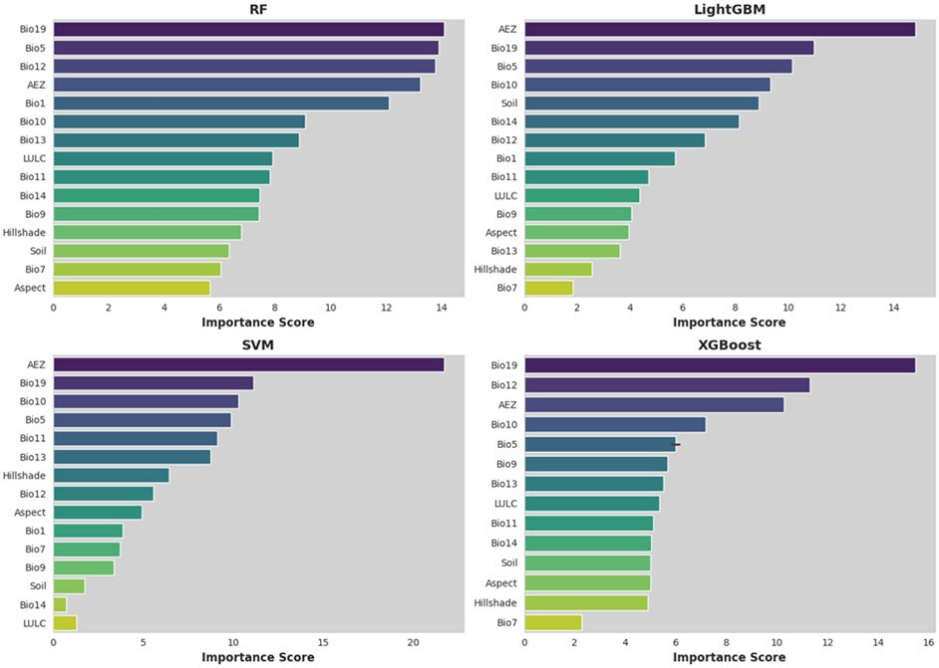
Relative variable importance in habitat suitability modeling of *A. mellifera*.

A cross‐model feature importance analysis using four machine learning algorithms, RF, SVM, LightGBM, and XGBoost, revealed both reliable and model‐specific patterns in the influence of environmental predictors for the species (Figure 4). Bio19 ranked highest in RF (14.09) and XGBoost (15.49), and second in LightGBM (10.98) and SVM (11.13), indicating its fundamental role in regulating seasonal water availability for the species in this study. AEZ thought the highest situation in LightGBM (14.83) and SVM (21.71) and ranked among the top four in RF (13.22) and XGBoost (10.28), reflecting its integrative capacity to represent regional climatic and edaphic conditions. Additionally, the mean temperature of the warmest quarter (Bio10) consistently ranked within the highest five in SVM, LightGBM, and XGBoost, while the maximum temperature of the warmest month (Bio5) showed high importance across RF (13.88), LightGBM (10.16), and SVM (9.87). In contrast, annual precipitation (Bio12) displayed model‐dependent variation ranking high in RF (13.78) and XGBoost (11.29) but lower in LightGBM (6.84) and SVM (5.57), suggesting different algorithmic responses to precipitation dynamics.

Significant model‐specific patterns included SVM's increased emphasis on AEZ, showing higher sensitivity to spatial variation, and LightGBM's higher ranking of soil features (importance score = 8.89), indicating its power in edaphic situations. Tree‐based models (RF, XGBoost) demonstrated greater consistency in bioclimatic hierarchies (e.g., Bio19 > Bio5 > Bio12), indicating their ability to represent hierarchical and nonlinear interactions. Variables such as aspect, hill shade, and temperature annual range (Bio7) consistently scored low across models, indicating their removal in favor of reduced dimensionality. Overall, Bio19 and AEZ are consistent among methods, with Bio5, Bio10, and Bio12 acting as context‐dependent predictors. Model selection has a significant influence on ecological inference, and properly aligning algorithms with ecological aims is critical for effective environmental modeling.

### 3.2 Performance Metrics of Machine Learning Algorithms

**FIGURE 5:**
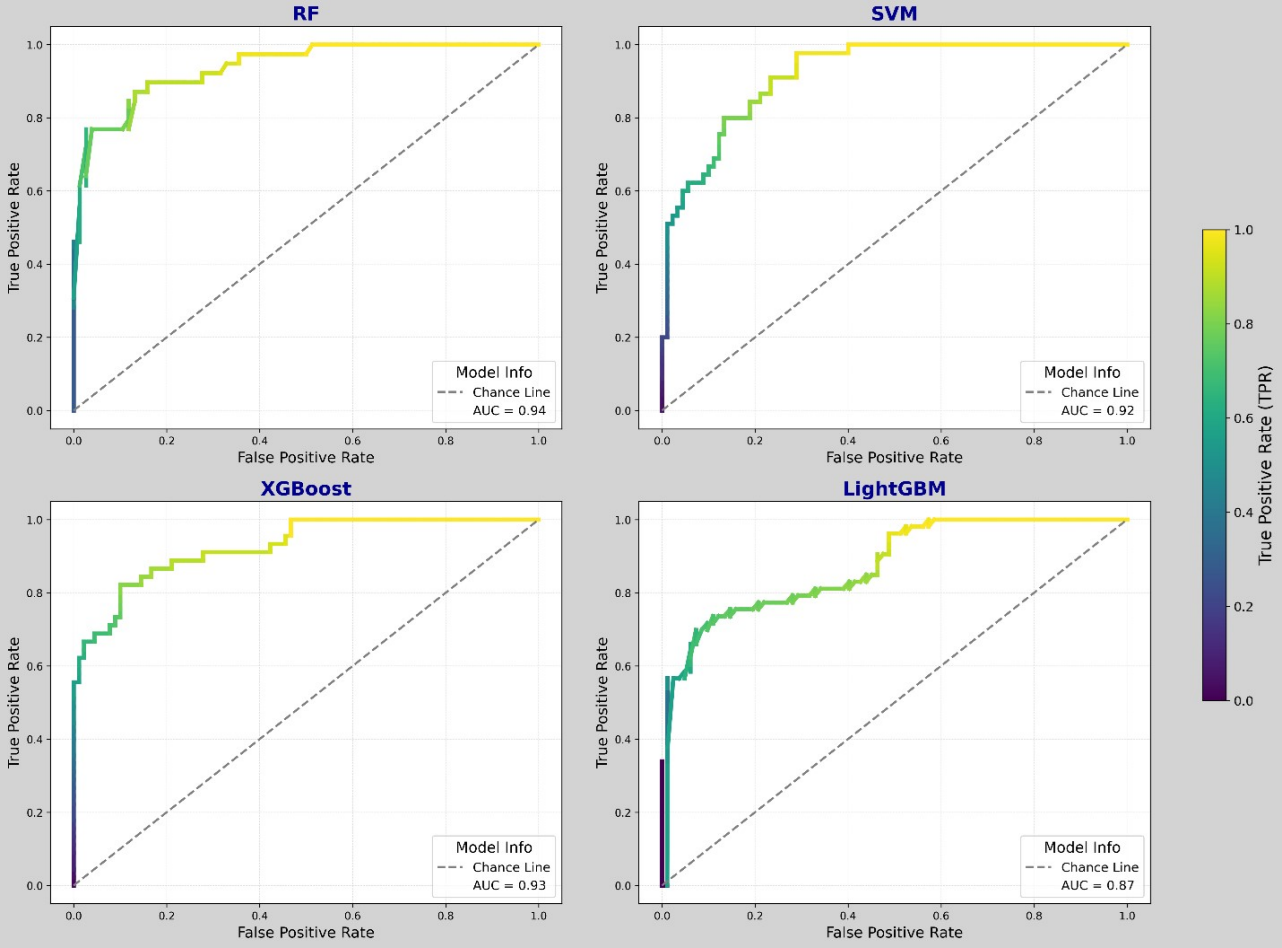
Comparison of the model of the mean ROC curve and AUC values.

**FIGURE 6:**
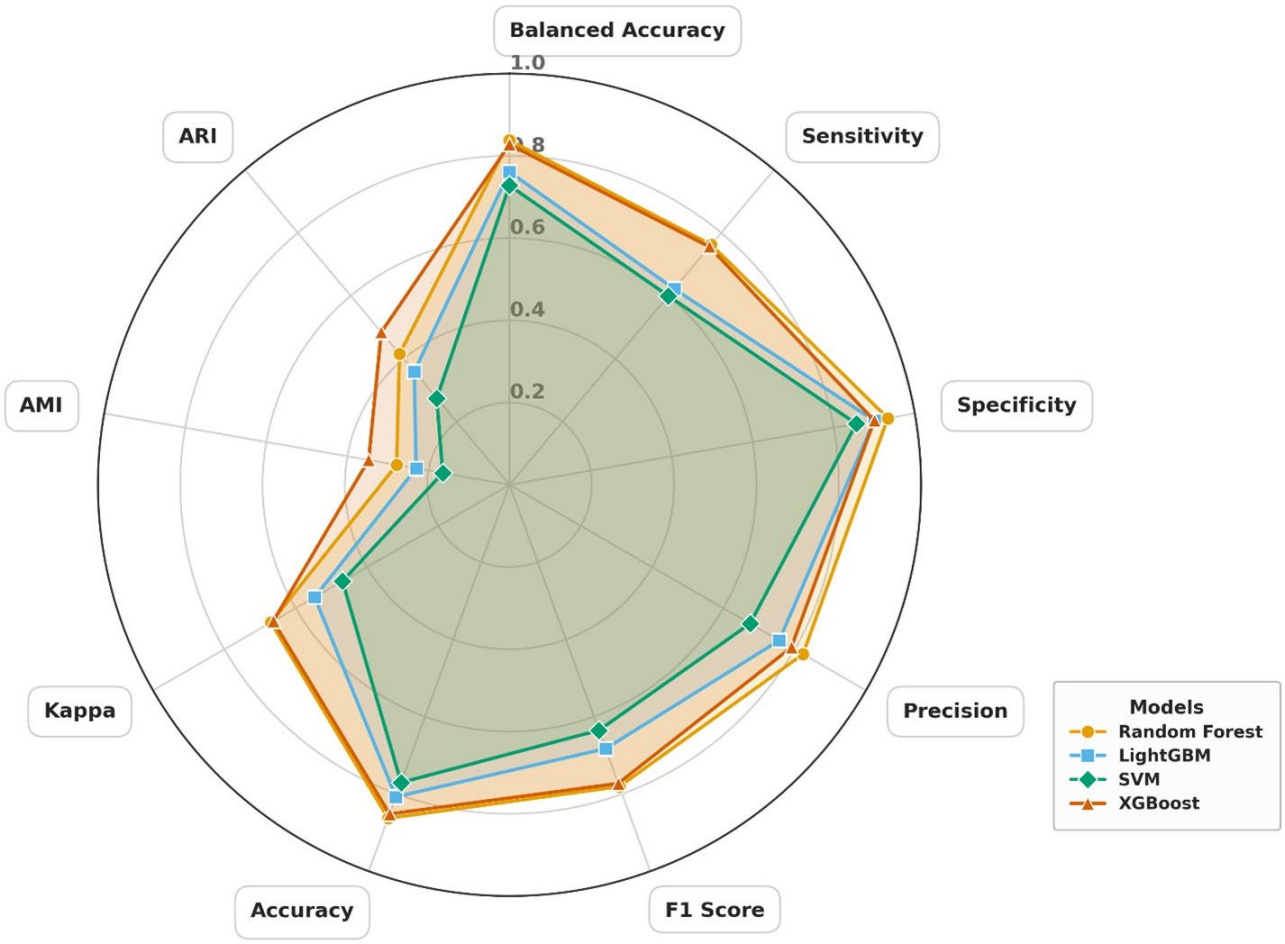
The classification, agreement, and clustering validity metrics among the four ML models.

Significant model performance differences were found in a quantitative evaluation of four machine learning algorithms, RF, XGBoost, LightGBM, and SVM, over 15 validity measures (Figures 5 and 6). Significant model performance showed differences among ML models with RF revealing higher discriminative power, peak values in specificity (0.93), precision (0.82), and AUC‐ROC (0.94), as well as strong balanced accuracy (0.84). Moreover, its clustering performance resulted in an AMI score of 0.28 and an ARI of 0.42. XGBoost emerged as the secondary classifier, achieving optimal clustering validity (AMI = 0.35; ARI = 0.49), minimal quantity disagreement (2), and competitive classification metrics such as balanced accuracy (0.83), F1 score (0.77), Kappa statistic (0.66), and AUC‐ROC (0.93), although with higher allocation disagreement (20) and exchange (18).

Following that, the LightGBM model demonstrated intermediate performance, with balanced accuracy (0.76), specificity (0.90), precision (0.76), accuracy (0.81), Kappa statistic (0.55), and AUC‐ROC (0.87). In contrast, SVM underperformed, recording the lowest values in 10/15 metrics: balanced accuracy (0.73), F1 score (0.64), accuracy (0.77), Kappa statistic (0.47), AMI (0.17), and ARI (0.28), as well as higher allocation disagreement (31) and exchange (26), with moderate specificity (0.856), and AUC‐ROC (0.92). The integration of these data displayed a strong performance hierarchy, with RF and XGBoost leading in classification robustness (AUC‐ROC superiority), agreement stability, and clustering coherence. While, LightGBM, good in specificity and accuracy, low in sensitivity‐related parameters. Despite achieving random classification levels, SVM had significant limits in discriminating and cluster alignment.

### 3.3 Habitat Suitability of *A. mellifera* Under Current Climate Conditions

**FIGURE 7:**
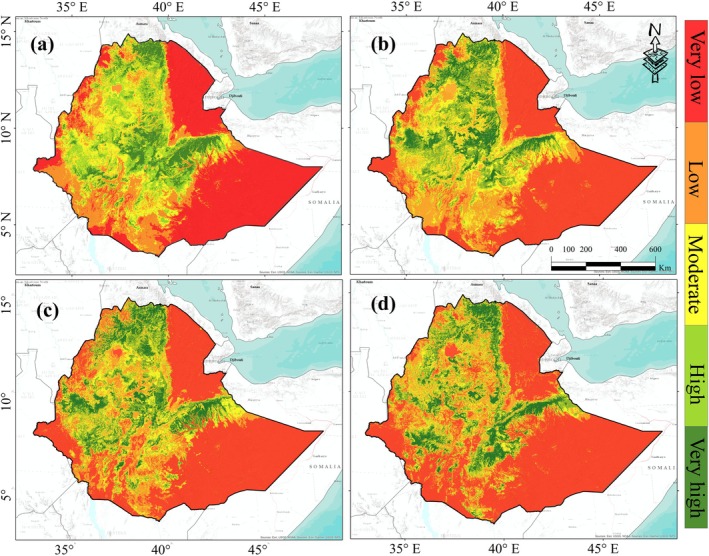
Current distribution of suitable areas for *Apis mellifera* across different machine learning models (a) Random Forest; (b) Supportive Vector Machine; (c) Extreme Gradient Boost; (d) Light Extreme Gradient Boost.

Under current climatic conditions, the suitability maps showed regional variances across ML models (Figure 7, Table 1, and Table S1) with the RF indicating 338,702.8 km^2^ (30.02%) of extremely suitable habitat, particularly in the Western Highlands. Of these, 12.01% had exceptionally high suitability, suggesting optimal environmental conditions. The SVM model projected high and very high appropriateness for 231,258 km^2^ (20.4%), divided into 84,209.51 km^2^ (7.43%) and 147,048.49 km^2^ (12.97%), respectively. XGBoost exhibited spatial patterns similar to RF but with clearer transitional zones. It classified 231,700.08 km^2^ (20.4%) as high and very high suitability, with 99,192.54 km^2^ (8.75%) in the very high category. LightGBM produced more alert and fragmented results, with 584,456.41 km^2^ (51.57%) classed as very low suitability and just 211,001.9 km^2^ (18.62%) as high and very high suitability combined.

**TABLE 1:** Current (ensemble) and future distribution of suitable areas for *Apis mellifera* under various climate change scenarios.

Suitability class	Current		Future distribution of suitable areas							
			ssp245 (2050)		ssp585 (2070)		ssp245 (2050)		ssp585 (2070)	
	km^2^	%	km^2^	%	km^2^	%	km^2^	%	km^2^	%
Very low	501,279.1	44.3	682,153	50.9	688,078	51.4	646,573	48.3	647,828	48.4
Low	211,729.6	18.7	236,416	17.7	304,205	22.7	204,187	15.3	239,460	17.9
Moderate	165,946.3	14.6	142,112	10.6	155,177	11.6	131,640	9.8	153,369	11.5
High	148,769.4	13.2	192,651	14.4	131,161	9.8	258,059	19.3	212,081	15.8
Very high	104,396.3	9.2	86,034	6.4	60,745	4.5	98,907	7.4	86,628	6.5

Abbreviations: km^2^, kilometer square; %, percentage.

In the present study, the Eastern Lowlands were consistently identified as unsuitable via all machine learning (ML) models. The Northern region of the nation had varying findings, with moderate to poor suitability ranging from 17.9% to 20.9% depending on the model. Moderate suitability was found in the Rift Valley, especially in the RF classification (15.11%). The Southern Regions demonstrated a range of suitability from high to poor; LightGBM had significant edge effects with complex spatial patterns, whereas RF generated smoother transitions. The ensemble model summary (Table 1) shows that 44.3% (501,279.1 km^2^) of the study region is classified as extremely poor suitability, with low and moderate suitability covering 18.7% and 14.6%, respectively. High and very high suitability, which are crucial for supporting healthy *A. mellifera* populations, account for 13.2% and 9.2%, respectively, or 22.4% of the habitat. These findings show the geographical complexity of acceptable habitats under present climatic conditions, with RF consistently showing the highest level of habitat suitability for the species.

### 3.4 Habitat Suitability of *A. mellifera* Under Future Climate Scenarios

**FIGURE 8:**
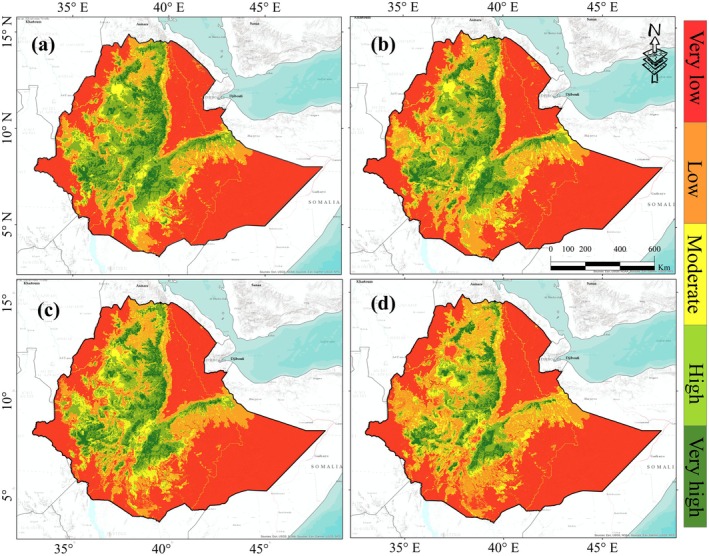
Future distribution of suitable areas for *A. mellifera* under various climate change scenarios (a) ssp245‐2050 (2041–2060); (b) ssp245‐2070 (2061–2080); (c) ssp585‐2050 (2041–2060); (d) ssp585‐2070 (2061–2080).

Future habitat suitability forecasts were developed based solely on bioclimatic variables and static topography predictors (see Section 2.7). Four scenarios generated by SSP245 and SSP585 for mid‐century (2050) and late‐century (2070) eras demonstrated consistent temporal and spatial decline in habitat suitability (Table 1, Figure 8). These projections revealed consistent temporal and spatial degradation of habitat suitability of *A. mellifera* in the study area. Additionally, future projections indicated that the suitability of the species will vary with agroecology. In the western highlands, SSP245‐2050 predicts considerable persistence of suitable habitat, with 98,907 km^2^ (7.38%) and 258,059 km^2^ (19.27%) classified as very high and high suitability, respectively. By 2070 under SSP245, these areas contract moderately to 86,628 km^2^ (6.47%), very high, and 212,081 km^2^ (15.83%) high suitability. Under the more severe SSP585 scenario, suitability becomes increasingly fragmented for the species. By 2050, very high suitability declines to 86,034 km^2^ (6.42%) and high suitability to 192,651 km^2^ (14.38%), with further reductions by 2070 to 60,745 km^2^ (4.54%) and 131,161 km^2^ (9.79%), respectively, indicating a 38.6% reduction in very high suitability relative to SSP245‐2050. The Central Highlands' moderate adaptability decreases significantly throughout scenarios, from 131,640 km^2^ (9.83%) under SSP245‐2050 to 153,369 km^2^ (11.45%) under SSP245‐2070, with additional losses by SSP585‐2070. High suitability also decreases significantly under SSP585 by 2070. The Eastern Lowlands remain mostly unsuitable, with extremely poor suitability rising from 646,573 km^2^ (48.27%) under SSP245‐2050 to 688,078 km^2^ (51.37%) under SSP585‐2070. Under future scenarios, the northern and southern areas have a higher proportion of low suitability classes, indicating a significant reduction of suitable habitat. These findings demonstrate *A. mellifera* 's vulnerability to climate change, with potential consequences for pollinator health and ecological services.

### 3.5 Habitat Diversity and Landscape Structure Analysis

**FIGURE 9:**
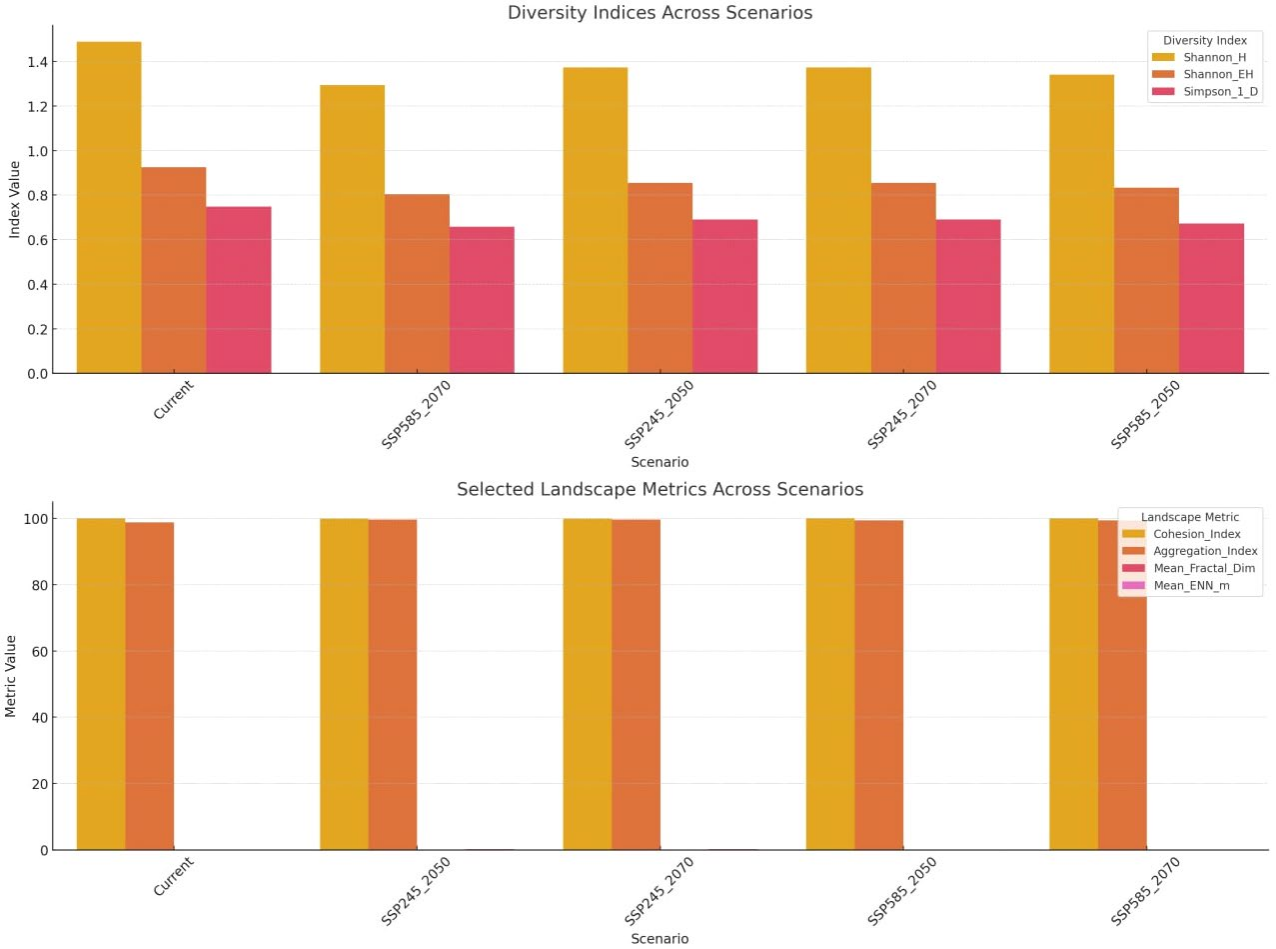
Habitat diversity and landscape structure analysis of *Apis mellifera*.

The result indicated that the diversity indices monitored changes in habitat richness and evenness, whereas landscape measures measured spatial structure and connectivity (Figure 9). Overall, the findings show a steady loss in habitat variety under future warming conditions. The Shannon Diversity Index (*H*′) was maximum under present circumstances (1.489), indicating ideal habitat dispersion, then gradually dropped to 1.294 during SSP585‐2070. SSP245 scenarios maintained equal intermediate values (1.374), showing generally steady suitability for relatively low emissions. Similarly, Shannon Equitability (EH) fell from 0.93 to 0.80 under SSP585‐2070 but remained higher under SSP245 (0.854). The Simpson Index (1–D) also declined from 0.75 to 0.66 under SSP585‐2070.

Landscape configuration measures revealed a decrease in Cohesion Index from 99.98 to 99.86 under SSP245‐2050, indicating increased habitat fragmentation. The Aggregation Index declined more significantly for SSP585 scenarios, indicating more scattered habitat patches. SSP245‐2070 increased mean fractal dimension from 0.17 to 0.20, indicating more patch irregularity. The average Euclidean Nearest Neighbor distance grew from 0.17 to 0.33 m under SSP245‐2050, whereas SSP585‐2070 had a lower value (0.134 m). Connectivity metrics indicated catastrophic losses, with the Connectance Index decreasing from 78,686.03 to less than 1.0 in future projections. Similarly, the Integral Index of Connectivity (IIC) and Probability of Connectivity decreased to 0.41 and 0.69, respectively, indicating a near‐complete collapse of landscape connection. SSP585‐2070 had a somewhat higher connection probability (0.69), but all subsequent values remained significantly lower than baseline levels (Figure 9).

## 4 Discussion

Beekeeping is crucial for global food security because it provides pollination services, and it also contributes significantly to rural livelihoods and national economies through honey, wax, and other hive products. In Ethiopia, apiculture provides a significant source of income for forest‐adjacent people while also contributing to biodiversity protection (FAO 2018). However, rising worldwide honey consumption, combined with climate change, habitat fragmentation, pesticide use, and novel pests, poses growing challenges to pollinator populations (Goulson et al. 2015; Teklewold et al. 2021). Temperature increases of 1°C by 2025 and 3°C by 2100 (IPCC 2018) are projected to drastically affect pest and disease dynamics, underscoring the importance of robust prediction frameworks for pollinator conservation (Lahlali et al. 2024; Proesmans et al. 2021).

This study addresses a gap by using a multi‐model machine learning framework based on Species Distribution Models (SDMs) to forecast current and future environmental suitability for *A. mellifera* in Ethiopia, including both climatic and non‐climatic factors. The findings are consistent with ecological theory, demonstrating that honeybee reactions to climate change are influenced by climate, landscape structure, and modeling approach (Elith and Leathwick 2009; Howard et al. 2014). Utilization of different algorithms and climate scenarios enhances forecast accuracy and captures a wide range of ecological reactions. While apiary‐based research provides essential local insights, it sometimes lacks spatial generalizability and mechanical linkages to environmental causes. Machine learning provides a scalable, data‐driven framework for modeling complex ecological systems and enables evidence‐based adaptation in apiculture (Fernandes et al. 2020). Furthermore, the significant genetic and ecological range of *A. mellifera* , especially in understudied tropical locations like Ethiopia, contributes complexity to vulnerability assessments.

### 4.1 Relative Variables and Model Performance

Advances in ecological acquiring data have improved species distribution models (SDMs), although these advances have also generated issues such as multicollinearity, which may influence model reliability and interpretability (Zhang et al. 2023). Ensemble machine learning techniques are often more resistant to multicollinearity than classic statistical models (Brown 2011). Temperature‐related variables (Bio2, Bio3, Bio6) and precipitation seasonality measures (Bio15, Bio17) showed severe multicollinearity (VIF > 900 and 564) and were eliminated to enhance model performance. This varies from earlier research (Assefa and Lemma 2022; Ma'moun et al. 2025; Rahimi et al. 2021), indicating the need for region‐specific variable selection. Ethiopia's Rift Valley and highland plateaus compress climatic gradients (Feleke et al. 2025; Gashaw et al. 2023), maintaining ecologically significant predictors with low collinearity, such as Agro‐Ecological Zones (AEZ; VIF < 3) and coldest quarter precipitation (Bio19). AEZ emerged as the most important predictor, suggesting landscape‐driven floral resource distribution, which is critical for Ethiopian apiculture (Bareke, Haile, et al. 2024; Bayissa et al. 2024; Gebremedhn et al. 2024). The significant contribution of Bio19 to tree‐based models (RF: 14.09%; XGBoost: 15.49%) indicates the relevance of dry‐season precipitation as a limitation on nectar availability, which is less prominent in temperate systems (Abou‐Shaara et al. 2017; Tabor and Koch 2021). Although other temperature‐related factors (Bio5, Bio10, Bio12) were shown to be context‐dependent, temperature remained a critical abiotic driver impacting insect growth, development, survival, fecundity, and dispersion (Coallier et al. 2025; Zapata‐Hernández et al. 2024). These findings suggest the need of regionally customized predictor selection in mountainous tropical systems, where human‐made landscapes significantly alter climate impacts.

The AUC‐ROC is a threshold‐independent statistic frequently utilized in ecological modeling (Leroy et al. 2016; Syphard and Franklin 2009). However, relying on a single metric may conceal biases related to class imbalance, sensitivity, and prevalence; thus, multiple classification and clustering metrics were used for comprehensive analysis (Konowalik and Nosol 2021). In this study, RF had the highest classification accuracy (AUC‐ROC: 0.94; specificity: 0.93) and efficiently captured hierarchical interactions (e.g., AEZ × Bio19) and non‐linear responses (Cutler et al. 2007; Gregorutti et al. 2017), while XGBoost outclassed at clustering (ARI: 0.49), accurately mapping fragmented suitability in diverse environments (Imani et al. 2025; Zheng et al. 2017). SVM proceeded poorly (sensitivity: 0.60), most likely due to difficulties in understanding high‐dimensional interactions in complicated terrain. LightGBM performed good, with a high sensitivity to slope (10.9% importance). Although all models attained AUC‐ROC ≥ 0.87 (Lobo et al. 2008; Shabani et al. 2018), this metric masked major performance differences, indicating the need for multi‐metric evaluation in conservation situations. Generally, RF produced the most balanced performance, combining strong classification and spatial patterning; XGBoost excelled in fragmentation detection but was less stable; LightGBM presented modest utility; and SVM was the least efficient. These findings are consistent with previous research on ensemble approaches in ecological modeling (Araújo and New 2007).

### 4.2 Climate Change Impact and Fragmentation of *A. mellifera*

Anthropogenic climate change affects pollinator populations through reducing flower biodiversity and foraging resources (Abou‐Shaara et al. 2017; Zapata‐Hernández et al. 2024). Climate regime shifts impact the phenology, abundance, and geographic distribution of nectar and pollen flora, reducing honeybee diet, colony health, and production (Walters et al. 2022). This study demonstrated that climate change will significantly affect the distribution and suitability of *A. mellifera* habitats throughout Ethiopia. Under this scenario, inter‐model variance was evident: the RF model predicted the largest region of high suitability (30.02%), predominantly in the Western Highlands, whereas LightGBM showed significant fragmentation, classifying 51.57% of the nation as extremely poor suitability. This inconsistency reveals model sensitivity to landscape arrangement. LightGBM's lower estimate of high suitability (18.62%) indicates significant edge effects in transitional ecotones, corresponding with fragmentation patterns found in xeric‐adapted *Apis florea* (Ma'moun et al. 2025), highlighting habitat fragility under climatic instability.

Ensemble models indicated significant geographical heterogeneity in present suitability and predicted significant deterioration under future climates. Under SSP585‐2070, very high‐suitability habitats fall by 51%, with a 46.2% loss in total high and very high suitability areas compared to current conditions. Landscape measurements show decreased patch diversity (Shannon Index: 1.489 → 1.294) and connection loss (IIC: 78,686 → < 1.0), suggesting threats to dispersion and metapopulation survival (Ntshanga et al. 2021). These findings are consistent with many studies that demonstrate pollinators as early responders to climate‐driven alterations in floral abundance (Goulson et al. 2015; Kerr et al. 2015; Scaven and Rafferty 2013). Thermal stress and unpredictable rainfall have caused the most severe habitat degradation in lowland and mid‐altitude regions, lowering floral abundance and synchrony. Given *A. mellifera's* vulnerability to such disturbances (Abou‐Shaara 2014), reduced floral supplies may increase absconding, lower honey productivity, and increase colony losses.

Topography and elevation shape species distribution under climate change, and Ethiopia's high‐elevation regions, Bale, Simien, Gera, and Sheka, may represent climate refugia under moderate and high emissions due to stable microclimates, rich plant diversity, and low human pressure, reflecting global upslope pollinator shifts (Fekadu et al. 2019; Lázaro et al. 2022). However, these refugia remain vulnerable, as Ethiopia's steep highland‐lowland terrain and current land use changes cause major barriers to species migration and long‐term survival (Aerts et al. 2019). In addition to climate‐related stresses, human activities such as deforestation, intensive grazing, and agricultural expansion increase pollinator vulnerability by causing habitat loss and fragmentation (Noman and Zafar 2023). The widespread use of agrochemicals in agricultural farming is another toxicity risk to honeybees, impairing foraging, navigation, and immunity (Tosi et al. 2017). Combined chemical and climate stresses may apply synergistic effects, intensifying colony losses, particularly in ecologically sensitive regions (Brunet and Fragoso 2024).

Research on pollinator habitat modeling indicates significant global declines in bee populations, primarily driven by climate change exceeding species‐specific thermal thresholds. Region‐specific projections reveal severe impacts: in Brazil, stingless bees (*Melipona* spp.) are projected to lose over 30% of their suitable habitat by 2050 (Lima and Marchioro 2021). Alongside, bee species in the eastern Amazon are expected to lose more than 85% of their suitable range, with native *Melipona* species suffering over 95% habitat loss (Giannini et al. 2020). Australian honeybee populations are approaching regional extirpation under intensifying environmental stressors (Tennakoon, Perera, and Jayasinghe 2024).

Ethiopia exhibits distinct ecological dynamics driving habitat suitability loss, including declining floral resources, such as a 19.09% habitat reduction for keystone forage plants like *Hypoestes forskaolii* (Gebremedhn et al. 2024). *Varroa destructor* infestations are projected to decline by 31.66% in cooler highlands (Gebremedhn et al. 2025), and recent epidemiological research further highlights the significance of *V. destructor* as a driver of colony loss in Ethiopia (Robi et al. 2023). Moreover, honeybee colony losses across Ethiopia are mainly attributed to queen failure, natural disasters (especially conflict), and poor pest management (Hailu et al. 2024). The interdependence of bees and key crops in Ethiopia demonstrates that *A. mellifera* and *Coffea arabica* (coffee) share closely linked climate dependencies within limited, vulnerable habitats (Abrha 2018). This highlights the need for integrated landscape management supporting both pollinator biodiversity and agricultural food security, as continued habitat degradation directly threatens pollination‐dependent production systems.

### 4.3 Limitations and Future Research Directions

The study has considerable limitations. Initially, future projections were based only on static topography features (elevation, slope, aspect, and hill shade) and bioclimatic factors. Dynamic predictors (land use, soil, and agro‐ecological zones) were omitted due to a lack of future data, resulting in an underestimation of overall habitat loss during concurrent land use change. Future research should replace static classifications with dynamic land‐use models like Land SHIFT (Verburg et al. 2002). Online occurrence data may result in spatial sampling bias (Phillips and Dudík 2008). Following that, a lack of socioeconomic and agrochemical data makes it difficult to evaluate anthropogenic factors. Using a single GCM (HadGEM3‐GC31‐LL) and two SSPs underrepresents uncertainty, according to (Eyring et al. 2016)., suggesting the need for multi‐model ensembles comprising several GCMs and SSPs. Multi‐species assessments and genomic screening of highland refugia (e.g., Bale, Simien) are required to discover community‐level thresholds and adaptive alleles (Klein et al. 2017).

### 4.4 Conservation and Agriculture Implications

The present findings recommend for immediate, wide‐ranging measures to conserve honeybee populations and agricultural resilience. Conservation efforts should prioritize the protection of microrefugia in sensitive areas, as well as the conservation of high‐elevation ecosystems that are protected from excessive warming. Furthermore, Ethiopia's Climate‐Resilient Green Economy framework should impose agrochemical inhibits in pollinator‐dependent zones to prevent pesticide‐driven colony collapse (Bareke, Roba, and Addi 2024). Restoring habitat connectivity is critical, as is planting drought‐tolerant native plants like *Vachellia tortilis* in degraded mid‐elevation ecotones to combat fragmentation and improve forage resilience (Muhammed and Elias 2024; Tesfay and Negash 2025). Real‐time stress monitoring in sensitive areas with IoT hive sensors can allow for faster reactions to climatic abnormalities (Zapata‐Hernández et al. 2024). Integrating pollinator corridors with forest carbon initiatives (e.g., REDD+) can help with financing while also harnessing climate funding and improving ecological synergy. Significantly, interventions require beekeeping communities, particularly women who dominate small‐scale apiculture, to promote fair adoption and local relevance. Mutually, these initiatives will address the predicted 46.2% reduction in high‐quality habitats, ensuring pollination for 85% of Ethiopia's crops and maintaining 2 million livelihoods endangered by fragmentation and climate uncertainty.

## 5 Conclusion

This study employed the integrated machine learning and landscape ecology techniques to evaluate the *A. mellifera* habitat suitability decline caused by climate change. The results showed, under SSP585‐2070, Ethiopia will lose 46.2% of its high‐suitability habitats, with very high suitability zones shrinking by 51% and connectivity indicators showing near‐total landscape fragmentation. The definite dominance of AEZ and Bio19 as predictors across ML models indicates the undoubted importance of seasonal water supply and topographic heterogeneity in maintaining viable populations. While ensemble modeling is critical for capturing Ethiopia's ecological complexity, with RF excelling in classification fidelity and XGBoost in clustering validity, the forecasts highlight existential risks to pollination services that sustain 85% of the nation's crops. Conservation success pivots on urgent interventions like formal protection of habitat refugia, landscape‐scale restoration of drought‐tolerant forage corridors featuring species like *Vachellia abyssinica*, and technological integration of mobile hive systems monitored through IoT sensors. These efforts must be embedded in national climate policy and co‐developed with beekeeping communities, particularly women, who represent most small‐scale apiarists. Without coordinated, science‐based action, Ethiopia faces biodiversity loss and the breakdown of agro‐ecological systems essential to food security. The current framework offers a scalable model for pollinator conservation in climate‐vulnerable, topographically complex regions worldwide.

## Author Contributions

**Diriba Tulu:** conceptualization (lead), data curation (lead), formal analysis (equal), investigation (equal), methodology (equal), project administration (equal), resources (equal), software (equal), supervision (lead), validation (equal), visualization (equal), writing – original draft (equal), writing – review and editing (lead). **Kalid Hassen Yasin:** data curation (equal), formal analysis (equal), investigation (equal), methodology (equal), resources (equal), software (equal), supervision (equal), validation (equal), visualization (equal), writing – review and editing (equal). **Tadele Bedo Gelete:** investigation (equal), data analysis, methodology (equal), resources, software (equal), validation (equal), visualization (equal), writing – review and editing (equal). **Beyan Ahmed:** investigation (equal), methodology (equal), validation (equal), visualization (equal), writing – review and editing (equal). **Dinaol Belina:** investigation (equal), methodology (equal), validation (equal), visualization (equal), writing – review and editing (equal).

## Funding

The authors have nothing to report.

## Ethics Statement

The authors have nothing to report.

## Conflicts of Interest

The authors declare no conflicts of interest.

## Supporting information


**Table S1:** Current habitat suitability classes of *Apis mellifera* under four ML models.
**Table S2:** Mathematical Formulas for Classification Metrics for ML model validation and evaluation.
**Table S3:** Lists of the environmental predictors used for modeling in the study.

## Data Availability

I confirm that the Data Availability Statement is included in the main file of my submission, and that access to all necessary data files is provided to editors and reviewers. The data supporting the findings of this study are openly available in Zenodo at https://doi.org/10.5281/zenodo.17069490.

## References

[ece373842-bib-0001] Abou‐Shaara, H. F. 2014. “The Foraging Behaviour of Honey Bees, *Apis mellifera* : A Review.” Saudi Journal of Biological Sciences 21: 101–105. 10.1016/j.sjbs.2013.12.004.

[ece373842-bib-0002] Abou‐Shaara, H. F. , A. A. Owayss , Y. Y. Ibrahim , and N. K. Basuny . 2017. “A Review of Impacts of Temperature and Relative Humidity on Various Activities of Honey Bees.” Insectes Sociaux 64: 455–463. 10.1007/S00040-017-0573-8.

[ece373842-bib-0003] Abrha, H. 2018. “Climate Change Impact on Coffee and the Pollinator Bee Suitable Area Interaction in Raya Azebo, Ethiopia.” Cogent Food & Agriculture 4: 1–13. 10.1080/23311932.2018.1564538.

[ece373842-bib-0004] Aerts, R. , F. Lerouge , and E. November . 2019. “Land Use Impacts on Vegetation in African Highlands: An Example From Ethiopia.” Science of the Total Environment 660: 836–845. 10.1016/j.scitotenv.2019.01.001.

[ece373842-bib-0005] Aiello‐Lammens, M. E. , R. A. Boria , A. Radosavljevic , B. Vilela , and R. P. Anderson . 2015. “spThin: An R Package for Spatial Thinning of Species Occurrence Records for Use in Ecological Niche Models.” Ecography 38: 541–545. 10.1111/ecog.01132.

[ece373842-bib-0006] AIRBUS . 2022. “Copernicus DEM: Copernicus Digital Elevation Model.” Product Handbook—Report AO/1‐9422/18/I‐LG Version 5.0.

[ece373842-bib-0007] Aligaz, M. A. , A. Bekele , and B. A. Bogale . 2024. “Predicting Climate Change Impact on the Habitat of Ethiopia's Spot‐Breasted Lapwing Using Ensemble Model.” Global Ecology and Conservation 54: e03139. 10.1016/j.gecco.2024.e03139.

[ece373842-bib-0008] Allouche, O. , A. Tsoar , and R. Kadmon . 2006. “Assessing the Accuracy of Species Distribution Models: Prevalence, Kappa and the True Skill Statistic (TSS).” Journal of Applied Ecology 43: 1223–1232.

[ece373842-bib-0009] Anselin, L. 1995. “Local Indicators of Spatial Association—LISA.” Geographical Analysis 27: 93–115. 10.1111/j.1538-4632.1995.tb00338.x.

[ece373842-bib-0010] Araújo, M. B. , and M. New . 2007. “Ensemble Forecasting of Species Distributions.” Trends in Ecology & Evolution 22: 42–47. 10.1016/j.tree.2006.09.010. 17011070

[ece373842-bib-0011] Asefa, M. , M. Cao , Y. He , E. Mekonnen , X. Song , and J. Yang . 2020. “Plant Diversity Ethiopian Vegetation Types, Climate and Topography.” Plant Diversity 42: 302–311. 10.1016/j.pld.2020.04.004. 33094201 PMC7567763

[ece373842-bib-0012] Assefa, A. , and M. Lemma . 2022. “Ecological Niche Modeling for Stingless Bees (Genus Melipona) in Waghemira and North Wollo Zones of Amhara Regional State, Ethiopia.” Scientific African 15: e01102. 10.1016/j.sciaf.2022.e01102.

[ece373842-bib-0013] Atsbha, T. , T. Gidey , H. Gebremedhin , and G. Bezabh . 2025. “Area Exclosure Improved Honey Bee Flora Diversity and Regeneration: The Case of Southern Tigrai, Northern Ethiopia.” Forest Science and Technology 21: 1–11. 10.1080/21580103.2025.2478890.

[ece373842-bib-0014] Austin, M. P. , and K. P. Van Niel . 2011. “Improving Species Distribution Models for Climate Change Studies: Variable Selection and Scale.” Journal of Biogeography 38: 1–8. 10.1111/j.1365-2699.2010.02416.x.

[ece373842-bib-0015] Bareke, T. , G. Haile , A. Addi , and K. Wakjira . 2024. “Ecological Suitability Analysis for Beekeeping Using GIS and AHP Model in Gedeo Zone of Southern Ethiopia.” Journal of Basic and Applied Research International 30: 9–23. 10.56557/jobari/2024/v30i18589.

[ece373842-bib-0016] Bareke, T. , K. Roba , and A. Addi . 2024. “Diversity of Bee Floral Resources and Honey Production Calendar in Ethiopia's Southwest Shoa Zone.” Advances in Agriculture 2024: 5428576. 10.1155/2024/5428576.

[ece373842-bib-0017] Bayissa, M. , L. Lauwers , F. Mitiku , D. C. de Graaf , and W. Verbeke . 2024. “System Mapping of the Production and Value Chain to Explore Beekeeping Potential in Southwest Ethiopia.” Insects 15: 106. 10.3390/insects15020106. 38392525 PMC10889247

[ece373842-bib-0018] BirdLife International . 2022. “ *Crithagra xantholaema* .” The IUCN Red List of Threatened Species 2022: e.T22720132A211581436.

[ece373842-bib-0019] Bivand, R. , J. Hauke , and T. Kossowski . 2013. “Computing the Jacobian in Gaussian Spatial Autoregressive Models: An Illustrated Comparison of Available Methods.” Geographical Analysis 45: 150–179. 10.1111/gean.12008.

[ece373842-bib-0020] Bladon, A. J. , P. F. Donald , N. J. Collar , et al. 2021. “Climatic Change and Extinction Risk of Two Globally Threatened Ethiopian Endemic Bird Species.” PLoS One 16: 1–17. 10.1371/journal.pone.0249633. PMC8133463 34010302

[ece373842-bib-0021] Brands, S. , S. Herrera , J. Fernández , and J. M. Gutiérrez . 2013. “How Well Do CMIP5 Earth System Models Simulate Present Climate Conditions in Europe and Africa?: A Performance Comparison for the Downscaling Community.” Climate Dynamics 41: 803–817. 10.1007/s00382-013-1742-8.

[ece373842-bib-0022] Breiman, L. 2001. “Random Forests.” Machine Learning 45: 5–32. 10.1023/A:1010933404324.

[ece373842-bib-0144] Brown, G. 2011. “Ensemble Learning.” In Encyclopedia of Machine Learning, edited by C. Sammut and G. I. Webb , 312–320. Springer. 10.1007/978-0-387-30164-8_252.

[ece373842-bib-0143] Brunet, J. , and F. P. Fragoso . 2024. “What are the Main Reasons for the Worldwide Decline in Pollinator Populations?” CABI Reviews 19, no. 1: 16. 10.1079/cabireviews.2024.0016.

[ece373842-bib-0025] Campbell, T. , K. W. Dixon , K. Dods , P. Fearns , and R. Handcock . 2020. “Machine Learning Regression Model for Predicting Honey Harvests.” Agriculture 10: 118. 10.3390/agriculture10040118.

[ece373842-bib-0026] Chen, T. , and C. Guestrin . 2016. “XGBoost: A Scalable Tree Boosting System.” In *Proceedings of the 22nd ACM SIGKDD International Conference on Knowledge Discovery and Data Mining* (pp. 785–794). 10.1145/2939672.2939785.

[ece373842-bib-0027] Clarke, D. , and D. Robert . 2018. “Predictive Modelling of Honey Bee Foraging Activity Using Local Weather Conditions.” Apidologie 49: 386–396. 10.1007/s13592-018-0565-3.

[ece373842-bib-0141] Coallier, N. , L. Perez , M. F. Franco , Y. Cuellar , and J. Vadnais . 2025. “Poor Air Quality Raises Mortality in Honey Bees, a Concern for All Pollinators.” Communications Earth & Environment 6: 126. 10.1038/s43247-025-02183-5. 39990959 PMC11845317

[ece373842-bib-0142] Cutler, D. R. , T. C. Edwards Jr. , K. H. Beard , et al. 2007. “Random Forests for Classification in Ecology.” Ecology 88, no. 11: 2783–2792. 10.1890/07-0539.1. 18051647

[ece373842-bib-0030] Deane‐mayer, A. Z. A. 2024. “aretEnsemble: Ensembles of Caret Models.” R Package Version 4.0.1. https://github.com/zachmayer/caretEnsemble. http://zachmayer.github.io/caretEnsemble/.

[ece373842-bib-0031] Demis, G. , T. Alemu , and S. Hassen . 2025. “Assessing the Diversity and Seasonal Availability of Honeybee Floral Resources in Amhara‐Sayint District, Northern Ethiopia.” Trees, Forests and People 20: 100886. 10.1016/j.tfp.2025.100886.

[ece373842-bib-0032] Deutsch, C. A. , J. J. Tewksbury , R. B. Huey , et al. 2008. “Impacts of Climate Warming on Terrestrial Ectotherms Across Latitude.” Proceedings of the National Academy of Sciences of the United States of America 105: 6668–6672. 10.1073/pnas.0709472105. 18458348 PMC2373333

[ece373842-bib-0033] Dormann, C. F. , J. Elith , S. Bacher , et al. 2013. “Collinearity: A Review of Methods to Deal With It and a Simulation Study Evaluating Their Performance.” Ecography 36: 27–46. 10.1111/j.1600-0587.2012.07348.x.

[ece373842-bib-0034] Drake, N. A. , S. Mackin , and J. J. Settle . 2006. “Mapping Land Cover and Estimating Land Cover Change Using SPOT Vegetation.” Remote Sensing of Environment 99: 357–367. 10.1016/j.rse.2005.08.016.

[ece373842-bib-0035] Ejigu, K. , A. Tadesse , and Y. Degu . 2021. “Foraging Ecology of *Apis mellifera simensis* : Implications for Pollinator Conservation in Ethiopia.” Journal of Insect Conservation 25: 789–798. 10.1007/s10841-021-00352-4.

[ece373842-bib-0036] El Alaoui, O. , and A. Idri . 2024. “An Empirical Evaluation of Ensemble Strategies in Habitat Suitability Modeling.” SN Computer Science 5: 501. 10.1007/s42979-024-02828-y.

[ece373842-bib-0037] Elith, J. , and J. R. Leathwick . 2009. “Species Distribution Models: Ecological Explanation and Prediction Across Space and Time.” Annual Review of Ecology, Evolution, and Systematics 40: 677–697. 10.1146/annurev.ecolsys.110308.120159.

[ece373842-bib-0140] Etherington, T. R. 2021. “Mahalanobis Distances for Ecological Niche Modelling and Outlier Detection: Implications of Sample Size, Error, and Bias for Selecting and Parameterising a Multivariate Location and Scatter Method.” PeerJ 9: e11436. 10.7717/peerj.11436. 34026369 PMC8121071

[ece373842-bib-0038] Evgeniou, T. , and M. Pontil . 2001. “Support Vector Machines: Theory and Applications.” Lecture Notes in Computer Science (Including Subseries Lecture Notes in Artificial Intelligence and Lecture Notes in Bioinformatics) 2049 LNAI, 249–257. 10.1007/3-540-44673-7_12.

[ece373842-bib-0039] Eyring, V. , S. Bony , G. A. Meehl , et al. 2016. “Overview of the Coupled Model Intercomparison Project Phase 6 (CMIP6) Experimental Design and Organization.” Geoscientific Model Development 9: 1937–1958. 10.5194/gmd-9-1937-2016.

[ece373842-bib-0040] FAO . 2018. “The Importance of Bees and Other Pollinators for Food and Agriculture (Report).” FAO's work on pollinators. https://www.fao.org/pollination/en.

[ece373842-bib-0041] FAO, IIASA . 2022. “Harmonized World Soil Database Version 2.0.”

[ece373842-bib-0042] FAO, IZSLT, Apimondia, CAAS . 2021. “Good Beekeeping Practices for Sustainable Apiculture.” 10.4060/cb5353en.

[ece373842-bib-0043] Fekadu, M. , H. Tveite , Y. Mamo , and B. Gessesse . 2019. “Ericaceous Vegetation of the Bale Mountains of Ethiopia Will Prevail in the Face of Climate Change.” Environmental Monitoring and Assessment 191: 135. 10.1007/s10661-019-7234-1.30734093

[ece373842-bib-0044] Feleke, H. G. , T. A. Amdie , F. Rasche , S. Y. Mersha , and C. Brandt . 2025. “Climate on the Edge: Impacts and Adaptation in Ethiopia's Agriculture.” Sustainability 17: 5119. 10.3390/su17115119.

[ece373842-bib-0045] Fernandes, A. C. M. , R. Q. Gonzalez , M. A. Lenihan‐Clarke , E. F. L. Trotter , and J. J. Arsanjani . 2020. “Machine Learning for Conservation Planning in a Changing Climate.” Sustainability (Switzerland) 12: 7657. 10.3390/su12187657.

[ece373842-bib-0046] Fick, S. E. , and R. J. Hijmans . 2017. “WorldClim 2: New 1‐km Spatial Resolution Climate Surfaces for Global Land Areas.” International Journal of Climatology 37: 4302–4315. 10.1002/joc.5086.

[ece373842-bib-0047] Food and Agriculture Organization . 2020. FAO Statistical Databases. Food and Agriculture Organization.

[ece373842-bib-0048] Franklin, J. 2010. “Moving Beyond Static Species Distribution Models in Support of Conservation Biogeography.” Diversity and Distributions 16: 321–330. 10.1111/j.1472-4642.2010.00641.x.

[ece373842-bib-0049] Gashaw, T. , G. B. Wubaye , A. W. Worqlul , et al. 2023. “Local and Regional Climate Trends and Variabilities in Ethiopia: Implications for Climate Change Adaptations.” Environmental Challenges 13: 100794. 10.1016/j.envc.2023.100794.

[ece373842-bib-0050] GBIF . 2025. “GBIF.org (13 February 2025) GBIF Occurrence Download.” 10.15468/dl.ay6xgb.

[ece373842-bib-0051] Gebrehiwet, M. , T. Haileselassie , F. Gadissa , and K. Tesfaye . 2019. “Genetic Diversity Analysis in *Plectranthus edulis* (Vatke) Agnew Populations Collected From Diverse Geographic Regions in Ethiopia Using Inter‐Simple Sequence Repeats (ISSRs) DNA Marker System.” Journal of Biological Research‐Thessaloniki 26: 7. 10.1186/s40709-019-0100-3. PMC6734312 31516863

[ece373842-bib-0052] Gebremedhn, H. , Y. Gebrewahid , G. Hadgu , and D. C. de Graaf . 2025. “Projecting the Impacts of Climate Change on Habitat Distribution of *Varroa destructor* in Ethiopia Using MaxEnt Ecological Modeling.” Science of the Total Environment 968: 178904. 10.1016/j.scitotenv.2025.178904. 39983493

[ece373842-bib-0053] Gebremedhn, H. , Y. Gebrewahid , G. G. Haile , et al. 2024. “Projecting the Impact of Climate Change on Honey Bee Plant Habitat Distribution in Northern Ethiopia.” Scientific Reports 14: 1–16. 10.1038/s41598-024-66949-3. 38982176 PMC11233736

[ece373842-bib-0054] Georgiades, P. , Y. Proestos , J. Lelieveld , and K. Erguler . 2023. “Machine Learning Modeling of *Aedes albopictus* Habitat Suitability in the 21st Century.” Insects 14: 1–19. 10.3390/insects14050447. PMC10231109 37233075

[ece373842-bib-0055] Giannini, T. C. , W. F. Costa , R. C. Borges , et al. 2020. “Climate Change in the Eastern Amazon: Crop‐Pollinator and Occurrence‐Restricted Bees Are Potentially More Affected.” Regional Environmental Change 20: 9. 10.1007/s10113-020-01611-y.

[ece373842-bib-0056] Goulson, D. , E. Nicholls , C. Botías , and E. L. Rotheray . 2015. “Bee Declines Driven by Combined Stress From Parasites, Pesticides, and Lack of Flowers.” Science (1979) 347: 1255957. 10.1126/science.1255957. 25721506

[ece373842-bib-0057] Gregorutti, B. B. , B. Michel , and P. P. Saint‐Pierre . 2017. “Correlation and Variable Importance in Random Forests.” Statistics and Computing 27: 659–678. 10.1007/s11222-016-9646-1.

[ece373842-bib-0058] Guisan, A. , W. Thuiller , and N. E. Zimmermann . 2013. “Predicting Species Distributions for Conservation Decisions.” Ecology Letters 16: 1424–1435. 10.1111/ele.12189. 24134332 PMC4280402

[ece373842-bib-0059] Hackett, T. D. , A. M. C. Sauve , K. P. Maia , et al. 2024. “Multi‐Habitat Landscapes Are More Diverse and Stable With Improved Function.” Nature 633: 114–119. 10.1038/s41586-024-07825-y. 39169178 PMC11374697

[ece373842-bib-0060] Hailu, T. G. , A. T. Atsbeha , K. Wakjira , and A. Gray . 2024. “High Rates of Honey Bee Colony Losses and Regional Variability in Ethiopia Based on the Standardised COLOSS 2023 Survey.” Insects 15: 376. 10.3390/insects15060376. 38921091 PMC11203459

[ece373842-bib-0061] Hao, T. , J. Elith , G. Guillera‐Arroita , and J. J. Lahoz‐Monfort . 2019. “A Review of Evidence About Use and Performance of Species Distribution Modelling Ensembles Like BIOMOD.” Diversity and Distributions 25: 839–852. 10.1111/ddi.12892.

[ece373842-bib-0062] Hesselbarth, M. H. K. , M. Sciaini , K. A. With , T. Wiegand , and J. Nowosad . 2019. “Landscapemetrics: An Open‐Source R Tool to Calculate Landscape Metrics.” Ecography 42: 1648–1657. 10.1111/ecog.04617.

[ece373842-bib-0063] Hijmans, R. J. , and J. van Etten . 2012. “raster: Geographic Data Analysis and Modeling.”

[ece373842-bib-0064] Howard, C. , P. A. Stephens , J. W. Pearce‐Higgins , R. D. Gregory , and S. G. Willis . 2014. “Improving Species Distribution Models: The Value of Data on Abundance.” Methods in Ecology and Evolution 5: 506–513. 10.1111/2041-210X.12184.

[ece373842-bib-0065] Hunde, T. A. , Y. Deneke , and B. H. Meressa . 2023. “Molecular Analyses of Mitochondrial DNA Reveal New Haplotypes and Lineages Within Ethiopian Honeybees ( *Apis mellifera* ).” International Journal of Tropical Insect Science 43: 1327–1338. 10.1007/s42690-023-01046-y.

[ece373842-bib-0066] Imani, M. , A. Beikmohammadi , and H. R. Arabnia . 2025. “Comprehensive Analysis of Random Forest and XGBoost Performance With SMOTE, ADASYN, and GNUS Under Varying Imbalance Levels.” Technologies (Basel) 13: 1–40. 10.3390/technologies13030088.

[ece373842-bib-0067] IPCC . 2018. “Global Warming of 1.5°C. An IPCC Special Report on the Impacts of Global Warming of 1.5°C Above Pre‐Industrial Levels and Related Global Greenhouse Gas Emission Pathways, in the Context of Strengthening the Global Response to the Threat of Climate Change.” In Press. 10.1002/9780470996621.ch50.

[ece373842-bib-0145] IPCC . 2021. “Climate Change 2021: The Physical Science Basis.” In Contribution of Working Group I to the Sixth Assessment Report of the Intergovernmental Panel on Climate Change, edited by V. Masson‐Delmotte , P. Zhai , A. Pirani , et al., 2391. Cambridge University Press. 10.1017/9781009157896.

[ece373842-bib-0068] IPCC . 2023. Climate Change 2023: Synthesis Report. Contribution of Working Groups I, II and III to the Sixth Assessment Report of the Intergovernmental Panel on Climate Change [Core Writing Team, H. Lee and J. Romero (eds.)]. IPCC. 10.59327/IPCC/AR6-9789291691647.

[ece373842-bib-0069] Jha, R. , A. Kanaujia , and K. K. Jha . 2022. “Wintering Habitat Modelling for Conservation of Eurasian Vultures in Northern India.” Nova Geodesia 2: 1–21.

[ece373842-bib-0070] Jiang, B. , S. He , H. Pan , and L. Li . 2022. “LightGBM for Climate Impact Modeling: An Interpretable Approach for Ecological Predictions.” Environmental Modelling and Software 149: 105317. 10.1016/j.envsoft.2022.105317.

[ece373842-bib-0071] Ke, G. , Q. Meng , T. Finley , et al. 2017. “LightGBM: A Highly Efficient Gradient Boosting Decision Tree.” Advances in Neural Information Processing Systems 30: 3146–3154.

[ece373842-bib-0072] Kerr, J. T. , A. Pindar , P. Galpern , et al. 2015. “Climate Change Impacts on Bumblebees Converge Across Continents.” Science (1979) 349: 177–180. 10.1126/science.aaa7031. 26160945

[ece373842-bib-0073] Klein, A.‐M. , B. E. Vaissiere , J. H. Cane , et al. 2017. “Importance of Pollinators in Changing Landscapes for World Crops.” Global Change Biology 13: 1–17. 10.1111/gcb.13656. PMC1702377 17164193

[ece373842-bib-0074] Knutti, R. , J. Sedláček , B. M. Sanderson , R. Lorenz , E. M. Fischer , and V. Eyring . 2017. “A Climate Model Projection Weighting Scheme Accounting for Performance and Interdependence.” Geophysical Research Letters 44: 1909–1918. 10.1002/2016GL072012.

[ece373842-bib-0075] Konowalik, K. , and A. Nosol . 2021. “Evaluation Metrics and Validation of Presence‐Only Species Distribution Models Based on Distributional Maps With Varying Coverage.” Scientific Reports 11: 1–15. 10.1038/s41598-020-80062-1. 33452285 PMC7811024

[ece373842-bib-0076] Krechemer, F. d. S. , and C. A. Marchioro . 2020. “Past, Present and Future Distributions of Bumblebees in South America: Identifying Priority Species and Areas for Conservation.” Journal of Applied Ecology 57: 1829–1839. 10.1111/1365-2664.13650.

[ece373842-bib-0077] Lahlali, R. , M. Taoussi , S.‐E. Laasli , et al. 2024. “Effects of Climate Change on Plant Pathogens and Host‐Pathogen Interactions.” Crop and Environment 3: 159–170. 10.1016/j.crope.2024.05.003.

[ece373842-bib-0078] Lázaro, A. , A. Nielsen , and Ø. Totland . 2022. “High Mountain Plants Interact With Pollinators Across Elevation and Latitude.” Ecology Letters 25: 842–856. 10.1111/ele.13946.

[ece373842-bib-0079] Lee‐Yaw, J. A. , M. Jenny L , S. Pironon , and S. N. Sheth . 2022. “Species Distribution Models Rarely Predict the Biology of Real Populations.” Ecography 2022: 1–16. 10.1111/ecog.05877.

[ece373842-bib-0080] Legesse, S. A. 2016. “The Outlook of Ethiopian Long Rain Season From the Global Circulation Model.” Environmental Systems Research 5: 1–16. 10.1186/s40068-016-0066-1.

[ece373842-bib-0081] Leroy, B. , C. N. Meynard , C. Bellard , F. Courchamp , and W. Thuiller . 2016. “Virtual Species Distribution Models Show How Altering the Range of Environmental Conditions in Calibration Affects Model Transferability.” Ecography 39: 601–613. 10.1111/ecog.01792.

[ece373842-bib-0082] Lima, V. P. , and C. A. Marchioro . 2021. “Brazilian Stingless Bees Are Threatened by Habitat Conversion and Climate Change.” Regional Environmental Change 21: 14. 10.1007/s10113-021-01751-9.

[ece373842-bib-0083] Liu, C.‐H. , M. White , and G. Newell . 2013. “Selecting Thresholds of Occurrence in the Prediction of Species Distributions.” Ecological Modelling 222: 486–492. 10.1016/j.ecolmodel.2010.12.013.

[ece373842-bib-0084] Lobo, J. M. , A. Jiménez‐valverde , and R. Real . 2008. “AUC: A Misleading Measure of the Performance of Predictive Distribution Models.” Global Ecology and Biogeography 17: 145–151. 10.1111/j.1466-8238.2007.00358.x.

[ece373842-bib-0085] MacInnis, G. , E. Normandin , and C. D. Ziter . 2023. “Decline in Wild Bee Species Richness Associated With Honey Bee ( *Apis mellifera* L.) Abundance in an Urban Ecosystem.” PeerJ 11: 1–26. 10.7717/peerj.14699. PMC9901307 36755869

[ece373842-bib-0086] Ma'moun, S. , R. Farag , K. Abutaleb , A. Metwally , A. Ali , and M. Yones . 2025. “Habitat Suitability Modelling for the Red Dwarf Honeybee ( *Apis florea* (Linnaeus)) and Its Distribution Prediction Using Machine Learning and Cloud Computing.” Neotropical Entomology 54: 18. 10.1007/s13744-024-01220-y. PMC11652650 39688784

[ece373842-bib-0087] Manes, S. , M. J. Costello , H. Beckett , et al. 2006. “Maximum Entropy Modeling of Species Geographic Distributions.” Biological Conservation 190: 231–259. 10.1016/j.ecolmodel.2005.03.026.

[ece373842-bib-0088] Martín, B. , J. González‐arias , and J. A. Vicente‐vírseda . 2021. “Machine Learning as a Successful Approach for Predicting Complex Spatio–Temporal Patterns in Animal Species Abundance.” Animal Biodiversity and Conservation 44: 289–301. 10.32800/abc.2021.44.0289.

[ece373842-bib-0089] Meixner, M. D. , M. A. Leta , N. Koeniger , and S. Fuchs . 2011. “The Honey Bees of Ethiopia Represent a New Subspecies of *Apis mellifera* – *Apis mellifera simensis* n. ssp.” Apidologie 42: 425–437. 10.1007/s13592-011-0007-y.

[ece373842-bib-0090] MoA . 2024. “Apiculture Resources Development and Protection Directive No. 1028/2024.”

[ece373842-bib-0091] Muhammed, A. , and E. Elias . 2024. “Landscape Dynamics and Biodiversity Conservation in the Ethiopian Highland: Assessing Impacts and Strategies for Sustainability.” In Landscape Architecture and Design, edited by S. Lousada . IntechOpen. 10.5772/intechopen.1006523.

[ece373842-bib-0092] NaBU . 2020. “NABU's Follow‐Up Biodiversity Assessment at the Kafa Biosphere Reserve, Ethiopia.”

[ece373842-bib-0093] Naimi, B. , and M. B. Araújo . 2016. “Sdm: A Reproducible and Extensible R Platform for Species Distribution Modelling.” Ecography 39: 368–375. 10.1111/ecog.01881.

[ece373842-bib-0094] Noman, Z. , and M. Zafar . 2023. “Fragile Balance: Interplay of Deforestation and Climate Change on Honeybee Population in Khyber Pakhtunkhwa, Pakistan.” Qlantic Journal of Social Sciences 4: 15–25. 10.55737/qjss.112446725.

[ece373842-bib-0095] Ntshanga, N. K. , S. Procheş , and J. A. Slingsby . 2021. “Assessing the Threat of Landscape Transformation and Habitat Fragmentation in a Global Biodiversity Hotspot.” Austral Ecology 46: 1052–1069. 10.1111/aec.13037.

[ece373842-bib-0096] Oksanen, J. , G. Blanchet , M. Friendly , et al. 2022. “vegan: Community Ecology Package.” R Package Version 2.6‐4.

[ece373842-bib-0097] Olson, D. M. , and E. Dinerstein . 2002. “The Global 200: Priority Ecoregions for Global Conservation.” Annals of the Missouri Botanical Garden 89: 199–224. 10.2307/3298564.

[ece373842-bib-0098] Phillips, S. J. , and M. Dudík . 2008. “Modeling of Species Distributions With Maxent: New Extensions and a Comprehensive Evaluation.” Ecography 31: 161–175. 10.1111/j.0906-7590.2008.5203.x.

[ece373842-bib-0099] Potts, S. G. , V. Imperatriz‐Fonseca , H. T. Ngo , et al. 2016. “Safeguarding Pollinators and Their Values to Human Well‐Being.” Nature 540: 220–229. 10.1038/NATURE20588. 27894123

[ece373842-bib-0100] Probst, P. , M. N. Wright , and A. L. Boulesteix . 2019. “Hyperparameters and Tuning Strategies for Random Forest.” Wiley Interdisciplinary Reviews: Data Mining and Knowledge Discovery 9: e1301. 10.1002/widm.1301.

[ece373842-bib-0101] Proesmans, W. , M. Albrecht , A. Gajda , et al. 2021. “Pathways for Novel Epidemiology: Plant–Pollinator–Pathogen Networks and Global Change.” Trends in Ecology & Evolution 36: 623–636. 10.1016/j.tree.2021.03.006. 33865639

[ece373842-bib-0102] R Core Team . 2025. R: A Language and Environment for Statistical Computing. R Core Team.

[ece373842-bib-0103] Rahimi, E. , S. Barghjelveh , and P. Dong . 2021. “Estimating Potential Range Shift of Some Wild Bees in Response to Climate Change Scenarios in Northwestern Regions of Iran.” Journal of Ecology and Environment 45: 14. 10.1186/s41610-021-00189-8.

[ece373842-bib-0104] Ramirez‐Diaz, J. , A. Manunza , T. A. de Oliveira , et al. 2025. “Combining Environmental Variables and Machine Learning Methods to Determine the Most Significant Factors Influencing Honey Production.” Insects 16: 278. 10.3390/insects16030278. 40266781 PMC11943014

[ece373842-bib-0105] Robi, D. T. , S. Temteme , M. Aleme , A. Bogale , A. Getachew , and E. Mendesil . 2023. “Epidemiology, Factors Influencing Prevalence and Level of Varroosis Infestation ( *Varroa destructor* ) in Honeybee ( *Apis mellifera* ) Colonies in Different Agroecologies of Southwest Ethiopia.” Parasite Epidemiology and Control 23: e00325. 10.1016/j.parepi.2023.e00325. 37711152 PMC10498395

[ece373842-bib-0106] Sahin Demirel, A. N. 2024. “Investigating the Impact of Climate Variables on the Organic Honey Yield in Turkey Using XGBoost Machine Learning.” Journal of the Science of Food and Agriculture 2024: 84–92. 10.1002/jsfa.13806. 39120002

[ece373842-bib-0107] Saupe, E. E. , A. Farnsworth , D. J. Lunt , N. Sagoo , K. V. Pham , and D. J. Field . 2019. “Climatic Shifts Drove Major Contractions in Avian Latitudinal Distributions Throughout the Cenozoic.” Proceedings of the National Academy of Sciences of the United States of America 116: 12895–12900. 10.1073/pnas.1903866116. 31182570 PMC6601418

[ece373842-bib-0108] Scaven, V. L. , and N. E. Rafferty . 2013. “Physiological Effects of Climate Warming on Flowering Plants and Insect Pollinators and Potential Consequences for Their Interactions.” Ecology Letters 16: 465–478. 10.1111/ele.12064. PMC3761068 24009624

[ece373842-bib-0109] Shabani, F. , L. Kumar , and M. Ahmadi . 2018. “Assessing Accuracy Methods of Species Distribution Models: AUC, Specificity, Sensitivity and the True Skill Statistic.” Global Journal of Human Social Science 18: 6–18.

[ece373842-bib-0110] Sibaja Leyton, M. , H. M. G. Lattorff , N. Kiatoko , and F. Requier . 2025. “Climate Effects on Honey Bees Can Be Mitigated by Beekeeping Management in Kenya.” Journal of Environmental Management 374: 123879. 10.1016/j.jenvman.2024.123879. 39765054

[ece373842-bib-0111] Singh, K. , M. Yadav , D. Barak , S. Bansal , and F. Moreira . 2025. “Machine‐Learning‐Based Frameworks for Reliable and Sustainable Crop Forecasting.” Sustainability (Switzerland) 17: 4711. 10.3390/su17104711.

[ece373842-bib-0112] Sirois‐Delisle, C. , and J. T. Kerr . 2018. “Climate Change‐Driven Range Losses Among Bumblebee Species Are Poised to Accelerate.” Scientific Reports 8: 1–10. 10.1038/s41598-018-32665-y. 30337544 PMC6194031

[ece373842-bib-0113] Sutherst, R. W. , G. F. Maywald , and A. S. Bourne . 2007. “Including Species Interactions in Risk Assessments for Global Change.” Global Change Biology 13: 1843–1859. 10.1111/j.1365-2486.2007.01396.x.

[ece373842-bib-0114] Syphard, A. D. , and J. Franklin . 2009. “Differences in Spatial Predictions Among Species Distribution Modeling Methods Vary With Species Traits and Environmental Predictors.” Ecography 32: 907–918. 10.1111/j.1600-0587.2009.05883.x.

[ece373842-bib-0115] Tabor, J. A. , and J. B. Koch . 2021. “Ensemble Models Predict Invasive Bee Habitat Suitability Will Expand Under Future Climate Scenarios in Hawai'i.” Insects 12: 443. 10.3390/insects12050443. 34067995 PMC8152285

[ece373842-bib-0116] Taubert, F. F. , R. Fischer , J. J. Groeneveld , et al. 2018. “Global Patterns of Tropical Forest Fragmentation.” Nature 554: 519–522. 10.1038/nature25508. 29443966

[ece373842-bib-0117] Teklewold, H. , M. Kassie , Z. Abro , K. Mulungu , and S. Sevgan . 2021. The Role of Pollination Services and Disrupting Cropping Patterns in Closing Nutrition Gap in Sub‐Saharan Africa. AgEcon Search.

[ece373842-bib-0118] Tennakoon, S. , A. Apan , and T. Maraseni . 2024. “Unravelling the Impact of Climate Change on Honey Bees: An Ensemble Modelling Approach to Predict Shifts in Habitat Suitability in Queensland, Australia.” Ecology and Evolution 14: 1–19. 10.1002/ece3.11300. PMC11024685 38638367

[ece373842-bib-0119] Tennakoon, T. , S. J. Perera , and H. Jayasinghe . 2024. “Climate‐Driven Shifts in Honeybee Distribution in Tropical Asia: A Maxent Modelling Approach.” Environmental Modelling and Software 165: 105705. 10.1016/j.envsoft.2024.105705.

[ece373842-bib-0120] Tesfay, H. M. , and M. Negash . 2025. “Ethiopia: Enhancing Landscape Connectivity Through Agroforests.” In Ecological Connectivity of Forest Ecosystems, 521–534. Springer Nature. 10.1007/978-3-031-82206-3_28.

[ece373842-bib-0121] Thuiller, W. , B. Lafourcade , R. Engler , and M. B. Araújo . 2009. “BIOMOD—A Platform for Ensemble Forecasting of Species Distributions.” Ecography 32: 369–373. 10.1111/j.1600-0587.2008.05742.x.

[ece373842-bib-0122] Tilahun, M. , A. Altaseb , and G. Bezabh . 2022. “Effect of Feeding *Acacia saligna* Pollen on *Apis mellifera* Adult Worker Bees in Northern Ethiopia.” International Journal of Tropical Insect Science 42: 3385–3393. 10.1007/s42690-022-00840-4.

[ece373842-bib-0123] Tosi, S. , G. Burgio , and J. C. Nieh . 2017. “A Common Neonicotinoid Pesticide, Thiamethoxam, Impairs Honey Bee Flight Ability.” Scientific Reports 7: 1201. 10.1038/s41598-017-01361-8. 28446783 PMC5430654

[ece373842-bib-0124] UN . 2024. The Sustainable Development Goals Report. United Nations.

[ece373842-bib-0125] van Etten, J. 2017. “gdistance: Distances and Routes on Geographical Grids.” R Package Version 1.2‐2.

[ece373842-bib-0126] Vapnik, V. N. 1999. The Nature of Statistical Learning Theory. Springer. 10.1007/978-1-4757-3264-1. 18252602

[ece373842-bib-0127] Verburg, P. H. , P. P. Schot , M. J. Dijst , and A. Veldkamp . 2002. “Land Use Change Modelling: Current Practice and Research Priorities.” GeoJournal 61: 309–324. 10.1023/B:GEJO.0000034735.59334.f7.

[ece373842-bib-0128] Voudiotis, G. , A. Moraiti , and S. Kontogiannis . 2022. “Deep Learning Beehive Monitoring System for Early Detection of the Varroa Mite.” Signals 3: 506–523. 10.3390/signals3030030.

[ece373842-bib-0129] Walters, J. , J. Zavalnitskaya , R. Isaacs , and Z. Szendrei . 2022. “Heat of the Moment: Extreme Heat Poses a Risk to Bee–Plant Interactions and Crop Yields.” Current Opinion in Insect Science 52: 100927. 10.1016/j.cois.2022.100927. 35500861

[ece373842-bib-0130] Wang, X. , Q. Xu , and J. Liu . 2023. “Determining Representative Pseudo‐Absences for Invasive Plant Distribution Modeling Based on Geographic Similarity.” Frontiers in Ecology and Evolution 11: 1–11. 10.3389/fevo.2023.1193602.38516293

[ece373842-bib-0132] Wickham, H. 2016. ggplot2: Elegant Graphics for Data Analysis. Springer. 10.1007/978-3-319-24277-4.

[ece373842-bib-0133] Wisz, M. S. , and A. Guisan . 2009. “Do Pseudo‐Absence Selection Strategies Influence Species Distribution Models and Their Predictions? An Information‐Theoretic Approach Based on Simulated Data.” BMC Ecology 9: 1–13. 10.1186/1472-6785-9-8. 19393082 PMC2680809

[ece373842-bib-0134] Yousefi, M. , S. Mohammadi , and A. Kafash . 2023. “Modeling Global Habitat Suitability and Environmental Predictor of Distribution of a Near Threatened Avian Scavenger at a High Spatial Resolution.” Frontiers in Ecology and Evolution 11: 1112962. 10.3389/fevo.2023.1112962.

[ece373842-bib-0135] Zapata‐Hernández, G. , M. Gajardo‐Rojas , M. Calderón‐Seguel , et al. 2024. “Advances and Knowledge Gaps on Climate Change Impacts on Honey Bees and Beekeeping: A Systematic Review.” Global Change Biology 30: e17219. 10.1111/gcb.17219. 38450832

[ece373842-bib-0136] Zhang, J. , F. Jiang , G. Li , et al. 2019. “Maxent Modeling for Predicting the Spatial Distribution of Three Raptors in the Sanjiangyuan National Park, China.” Ecology and Evolution 9: 6643–6654. 10.1002/ece3.5243. 31236249 PMC6580265

[ece373842-bib-0137] Zhang, W. , X. Gu , L. Hong , L. Han , and L. Wang . 2023. “Comprehensive Review of Machine Learning in Geotechnical Reliability Analysis: Algorithms, Applications and Further Challenges.” Applied Soft Computing 136: 110066. 10.1016/j.asoc.2023.110066.

[ece373842-bib-0138] Zheng, S. , J. Wu , M. Pan , X. Xu , and H. Duan . 2017. “XGBoost Model for Predicting Compound Bioactivity.” Molecular Informatics 36: 1600085. 10.1002/minf.201600085.

[ece373842-bib-0139] Zurell, D. , J. Franklin , C. König , et al. 2020. “A Standard Protocol for Reporting Species Distribution Models.” Ecography 43: 1261–1277. 10.1111/ecog.04960.

