# Supplementary material for: Machine Learning and Geospatial Modeling of Climate Change Impacts on Ethiopian Honeybees for Conservation and Resilient Agriculture
Source: Ecol Evol. 2026 Jun 14;16(6):e73842. doi: 10.1002/ece3.73842 (PMC13265244; doi:10.1002/ece3.73842)
Supplement: Supplementary file 1 — Table S1: Current habitat suitability classes of Apis mellifera under four ML models. Table S2: Mathematical Formulas for Classification Metrics for ML model validation and evaluation. Table S3: Lists of the environmental predictors used for modeling in the study. [file ECE3-16-e73842-s001.docx]

Table S1. Current habitat Suitability classes of A. mellifera under four ML models

| Suitability Classes | Machine Learning Models | | | | | | | |
| --- | --- | --- | --- | --- | --- | --- | --- | --- |
|  | Random Forest | | XGBOOST | | Light-GBM | | SVM | |
|  | Km2 | % | Km2 | % | Km2 | % | Km2 | % |
| Very Low | 416824.7 | 36.95 | 531,664.44 | 46.91 | 584,456.41 | 51.57 | 472,170.98 | 41.66 |
| Low | 202415.9 | 17.94 | 202,895.78 | 17.9 | 204,867.88 | 18.08 | 236,738.78 | 20.89 |
| Moderate | 170433.5 | 15.11 | 167,097.95 | 14.74 | 133,039.52 | 11.74 | 193,214.23 | 17.05 |
| High | 203190.4 | 18.01 | 132,507.54 | 11.69 | 112,331.26 | 9.91 | 147,048.49 | 12.97 |
| Very High | 135512.4 | 12.01 | 99,192.54 | 8.75 | 98,670.64 | 8.71 | 84,209.51 | 7.43 |

Table 2. Mathematical Formulas for Classification Metrics for ML model validation and evaluation

| **Metric** | **Equation** | **Citation** |
| --- | --- | --- |
| Balanced Accuracy | BalAcc = $\frac{1}{2} (\frac{TP}{TP+FN} +\frac{TN}{TN+FP} )$ | Brodersen et al. (2010), |
| Sensitivity (Recall) | Sensitivity = $\frac{TP}{TP+FN}$ | Fawcett (2006), |
| Specificity | Specificity = $\frac{TN}{TN+FP}$ | Fawcett (2006), |
| Precision | Precision = $\frac{TP}{TP+FP}$ | Fawcett (2006), |
| F1-Score | F1 = 2 x$(\frac{\mathrm{Precision}\times\mathrm{Recall}}{\mathrm{Precision}+\mathrm{Recall}} +\frac{2TP}{2TP+FP+FN} )$ | Sasaki (2007), |
| AUC-ROC | AUC = $\int_{0}^{1} \mathrm{TPR}(FPR)d(\mathrm{FPR})$  where TPR=TPTP+FNTPR=TP+FNTP​, FPR=FPFP+TNFPR=FP+TNFP​. | Hanley & McNeil (1982), |
| Cohen’s Kappa (κ) | *Κ* = $\frac{po-pe}{1-pe}$ ; where  po = $\frac{TP+FN}{N}$(observed agreement), pe = $\frac{(\mathrm{TP}+\mathrm{FN})(\mathrm{TP}+\mathrm{FP})+(\mathrm{FP}+\mathrm{TN})(\mathrm{FN}+\mathrm{TN})}{N^{2}}$(chance agreement). | Cohen (1960) |
| Allocation Disagreement (AD) | AD = 2×min (FP, FN) | Pontius & Millones (2011), |
| Quantity Disagreement (QD) | QD = \|FP - FN\| | Pontius & Millones (2011), |
| Adjusted Mutual Information (AMI) | AMI = $\frac{\mathrm{MI}(U,V)-E[\mathrm{MI}]}{max(H(U), H(V)) - E[MI]}$ | Vinh et al. (2010), JMLR |
| Adjusted Rand Index (ARI) | ARI = $\frac{RI - E[RI]}{max(RI) - E[RI]}$ where RI= $\frac{a+b}{{C_{2}}^{N}}$ | Hubert & Arabie (1985), |

*The symbol descriptions include TP for True Positives, TN for True Negatives, FP for False Positives, FN for False Negatives, and N as the total number of samples calculated by TP + TN + FP + FN. TPR stands for True Positive Rate, also known as Recall, while FPR denotes the False Positive Rate. The term “pₒ represents observed agreement, and pₑ refers to the expected agreement by chance. U and V are two cluster label assignments. MI stands for Mutual Information, and H(U) represents the entropy of partition U. RI denotes the Rand Index. The symbol a indicates the number of pairs in the same cluster in both partitions, and b signifies the number of pairs in different clusters in both partitions.*

Table 3. Lists of the environmental predictors used for modeling in the Study

| Name | Symbol | Description | Unit | Source |
| --- | --- | --- | --- | --- |
| Annual Mean Temperature | BIO1 | Mean annual temperature | °C | Fick & Hijmans, 2017 |
| Mean Diurnal Range | BIO2 | Mean of monthly (max temp - min temp) | °C | Fick & Hijmans, 2017 |
| Isothermality | BIO3 | BIO2/BIO7 × 100 | % | Fick & Hijmans, 2017 |
| Temperature Seasonality | BIO4 | Standard deviation of temperature × 100 | Unitless | Fick & Hijmans, 2017 |
| Max Temperature of Warmest Month | BIO5 | Highest mean temperature in the warmest month | °C | Fick & Hijmans, 2017 |
| Min Temperature of Coldest Month | BIO6 | Lowest mean temperature in the coldest month | °C | Fick & Hijmans, 2017 |
| Temperature Annual Range | BIO7 | BIO5 - BIO6 | °C | Fick & Hijmans, 2017 |
| Mean Temperature of Wettest Quarter | BIO8 | Mean temp during the wettest quarter | °C | Fick & Hijmans, 2017 |
| Mean Temperature of Driest Quarter | BIO9 | Mean temp during driest quarter | °C | Fick & Hijmans, 2017 |
| Mean Temperature of Warmest Quarter | BIO10 | Mean temp during the warmest quarter | °C | Fick & Hijmans, 2017 |
| Mean Temperature of Coldest Quarter | BIO11 | Mean temp during coldest quarter | °C | Fick & Hijmans, 2017 |
| Annual Precipitation | BIO12 | Total annual precipitation | mm | Fick & Hijmans, 2017 |
| Precipitation of Wettest Month | BIO13 | Precipitation during the wettest month | mm | Fick & Hijmans, 2017 |
| Precipitation of Driest Month | BIO14 | Precipitation during the driest month | mm | Fick & Hijmans, 2017 |
| Precipitation Seasonality (CV) | BIO15 | Coefficient of variation of monthly precipitation | % | Fick & Hijmans, 2017 |
| Precipitation of Wettest Quarter | BIO16 | Precipitation during the wettest quarter | mm | Fick & Hijmans, 2017 |
| Precipitation of Driest Quarter | BIO17 | Precipitation during the driest quarter | mm | Fick & Hijmans, 2017 |
| Precipitation of Warmest Quarter | BIO18 | Precipitation during the warmest quarter | mm | Fick & Hijmans, 2017 |
| Precipitation of Coldest Quarter | BIO19 | Precipitation during the coldest quarter | mm | Fick & Hijmans, 2017 |
| Elevation | DEM | Digital elevation model | m | AIRBUS, 2022 |
| Slope | SLOPE | Steepness of terrain | Degrees | AIRBUS, 2022 |
| Aspect | ASPECT | Direction terrain faces | Degrees | AIRBUS, 2022 |
| Terrain Roughness | TRI | Index of terrain variability | Unitless | AIRBUS, 2022 |
| Soil pH | SOIL_PH | Acidity/alkalinity of soil | pH | Hengl et al., 2017 |
| Organic Carbon | SOIL_OC | Soil organic carbon content | g/kg | Hengl et al., 2017 |
| Sand Fraction | SAND | Percentage of sand in soil | % | Hengl et al., 2017 |
| Silt Fraction | SILT | Percentage of silt in soil | % | Hengl et al., 2017 |
| Clay Fraction | CLAY | Percentage of clay in soil | % | Hengl et al., 2017 |
| Land Use/Land Cover | LULC | Land cover types and usage | Categorical | Li et al., 2017 |
| Hillshade | HLS | terrain surface | Unitless | AIRBUS, 2022 |
| Agro-Ecological Zones | AEZ | Regional ecological classifications | Categorical | MoA, 2024; FAO, 2020 |
